# The plastid genome of some eustigmatophyte algae harbours a bacteria-derived six-gene cluster for biosynthesis of a novel secondary metabolite

**DOI:** 10.1098/rsob.160249

**Published:** 2016-11-30

**Authors:** Tatiana Yurchenko, Tereza Ševčíková, Hynek Strnad, Anzhelika Butenko, Marek Eliáš

**Affiliations:** 1 Faculty of Science, Department of Biology and Ecology, Life Science Research Centre, University of Ostrava, Chittussiho 10, 710 00 Ostrava, Czech Republic; 2 Faculty of Science, Institute of Environmental Technologies, University of Ostrava, Chittussiho 10, 710 00 Ostrava, Czech Republic; 3 Institute of Molecular Genetics of the ASCR, v. v. i., Prague, Czech Republic

**Keywords:** Eustigmatophyceae, horizontal gene transfer, plastid genome, secondary metabolism, sugar phosphate cyclase superfamily, UbiA superfamily

## Abstract

Acquisition of genes by plastid genomes (plastomes) via horizontal gene transfer (HGT) seems to be a rare phenomenon. Here, we report an interesting case of HGT revealed by sequencing the plastomes of the eustigmatophyte algae *Monodopsis* sp. MarTras21 and *Vischeria* sp. CAUP Q 202. These plastomes proved to harbour a unique cluster of six genes, most probably acquired from a bacterium of the phylum Bacteroidetes, with homologues in various bacteria, typically organized in a conserved uncharacterized putative operon. Sequence analyses of the six proteins encoded by the operon yielded the following annotation for them: (i) a novel family without discernible homologues; (ii) a new family within the superfamily of metallo-dependent hydrolases; (iii) a novel subgroup of the UbiA superfamily of prenyl transferases; (iv) a new clade within the sugar phosphate cyclase superfamily; (v) a new family within the xylose isomerase-like superfamily; and (vi) a hydrolase for a phosphate moiety-containing substrate. We suggest that the operon encodes enzymes of a pathway synthesizing an isoprenoid–cyclitol-derived compound, possibly an antimicrobial or other protective substance. To the best of our knowledge, this is the first report of an expansion of the metabolic capacity of a plastid mediated by HGT into the plastid genome.

## 1. Background

Eustigmatophytes are a small yet expanding class of stramenopile algae (ochrophytes), nowadays perceived as a particularly interesting target for biotechnological research and exploitation thanks to the ability of some of the members to produce high amounts of lipid substances as energy and carbon reserves, promising for biofuel production [1,2]. This has driven research on eustigmatophytes into the genomics era, resulting in a number of completed genome sequencing projects [3–6]. They, however, targeted only members of the genus *Nannochloropsis*, recently divided into two separate (but related) genera: *Nannochloropsis* (*sensu stricto*) and *Microchloropsis* [7]. These projects have also yielded sequences of organellar (both plastid and mitochondrial) genomes, which are now available for nearly all species of the traditionally circumscribed genus *Nannochloropsis* [3,8,9].

**Figure 1.**
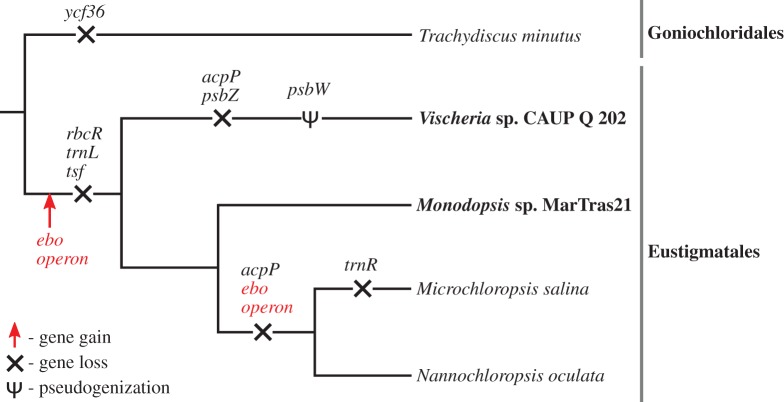
Schematic phylogeny of eustigmatophytes showing the position of species with sequenced plastid genomes. The taxa with plastomes sequenced in this study are highlighted in bold. For the genera *Nannochloropsis* and *Microchloropsis*, only the type species are shown for simplicity, although plastomes have been sequenced for a number of other closely related species or strains. The topology of the tree reflects a robustly resolved phylogeny based on the 18S rRNA gene [14]. Evolutionary events impacting the gene content of eustigmatophyte plastid genomes are mapped onto the tree based on the most parsimonious interpretation of the pattern of the gene presence/absence (provided in the electronic supplementary material, table S1).

However, recent surveys revealed a surprising phylogenetic diversity of the class Eustigmatophyceae [10,11]. This prompted us to initiate a more comprehensive investigation of eustigmatophytes at the genomic level. The first fruit of these efforts was sequencing the plastid genome (plastome for short) of *Trachydiscus minutus* [12], a member of a recently recognized major eustigmatophyte clade, Goniochloridales, which is deeply diverged from the group comprising ‘traditional’ eustigmatophytes including *Nannochloropsis* [10,13]. More recently, we reported results of a comparative analysis of eustigmatophyte mitochondrial genomes, including newly sequenced genomes of three phylogenetically diverse species, *T. minutus*, *Vischeria* sp. CAUP Q 202 and *Monodopsis* sp. MarTras21 [14]. In this study, we describe complete plastome sequences of *Vischeria* sp. CAUP Q 202 and *Monodopsis* sp. MarTras21 (for a schematic tree showing the relative phylogenetic position of the different eustigmatophytes discussed in this study see figure 1). As discussed below, analyses of these two genomes uncovered an unexpected evolutionary event that nominates the respective eustigmatophytes to a position of highly interesting candidates for further biochemical investigations with promise towards biotechnological applications.

Plastomes have evolved from the genome of the cyanobacterial progenitor that gave rise to plastids through the process of primary endosymbiosis dated between 1.7 and 0.9 billion years ago [15]. The primary mode of genome evolution in plastids was reduction, partly outright gene loss, partly relocation of the genes to the nuclear genome of the host eukaryotic cell via the process of endosymbiotic gene transfer [16]. As a result, the most gene-rich extant plastomes, found in red algae (rhodophytes), contain only up to around 250 protein-coding genes [17]. However, an opposite process, i.e. gene gain, has also been documented as a factor shaping the plastome gene repertoire. This includes not only emergence of extra genes by gene duplication [18,19], but notably also horizontal gene transfer (HGT) from foreign genomes.

The number of known instances of plastid genes gained by HGT is growing, but is still quite limited. The first documented HGT cases affecting plastid genomes were replacements of original cyanobacterial genes: of the RuBisCO operon by a Proteobacteria-derived operon in red algae [20] and of the *rpl36* gene in haptophytes and cryptophytes by an isoform of the same gene gained from a bacterial donor [21]. Nevertheless, HGT can result in gains of new genes not normally present in plastomes. Interestingly, the majority of known foreign genes in plastid genomes of various algal groups encode proteins operating on DNA or RNA, including DNA polymerases, recombinases, integrases, reverse transcriptases or maturases [18,22–27]. The significance of these genes for the functioning of the plastid genome and plastid as a whole remains unclear, but many of these acquisitions apparently relate to the activity of various mobile genetic elements, including group II introns [24,28], transposons [26] and plasmids [27]. Plastomes of several angiosperms were shown to harbour segments derived from mitochondrial genomes [29,30], but the functional significance of these elements is also unknown.

Much rarer are the examples of HGT-derived plastid genes conferring a clear metabolic function. We are aware of only one such case, specifically the presence of the cluster of the *leuC* and *leuD* genes in the plastome of the red alga *Gracilaria tenuistipitata* var. *liui* [31]. These genes encode the large and small subunits of 3-isopropylmalate dehydratase, an enzyme involved in leucine biosynthesis, and were apparently moved to the plastome of the *G. tenuistipitata* lineage by HGT from a eubacterium. In contrast to other red algae, *G. tenuistipitata* may lack a nuclear genome-encoded plastid-targeted 3-isopropylmalate dehydratase, and the authors suggested that this loss has been complemented by the acquisition of the *leuC* and *leuD* genes by the plastome. However, to the best of our knowledge, there has been no documented case of an acquisition of genes by a plastid genome that would expand the metabolic capacity of the respective plastid.

We were, therefore, highly surprised to find out that the newly sequenced plastid genomes of *Vischeria* sp. CAUP Q 202 and *Monodopsis* sp. MarTras21 contain a novel six-gene cluster encoding proteins homologous to various enzymes from bacteria. Orthologues of these genes could not be identified in other plastomes, including those previously reported from eustigmatophytes (i.e. those from *T. minutus* and *Nannochloropsis*/*Microchloropsis* spp.). This finding points to a peculiar case of HGT from bacteria to the plastid genome of a eustigmatophyte lineage that has endowed these algae with a new metabolic pathway. Our sequence and phylogenetic analyses described below indicated that the gene cluster, a putative novel operon, is widespread in many groups of bacteria and that at least five of the proteins encoded by the operon represent novel enzymes of different (super)families. While it is impossible to make a specific prediction about the function of these enzymes and the operon as a whole, the data available suggest that the eustigmatophytes and bacteria endowed with the operon may have the capacity to synthesize a novel secondary metabolite that may prove to be a practically interesting bioactive compound.

## 2. Material and methods

### 2.1. Sequencing and assembly of plastid genomes of *Vischeria* sp. CAUP Q 202 and *Monodopsis* sp. MarTras21

Obtaining draft genome sequence data from *Vischeria* sp. CAUP Q 202 and *Monodopsis* sp. MarTras21 was described previously [14]. Briefly, 454 and Illumina reads (in the case of *Vischeria* sp.) or Illumina reads only (in the case of *Monodopsis* sp.) generated from total DNA isolated from the respective algal cultures were assembled to obtain a mixed assembly of the nuclear and organellar genomes. Scaffolds representing the plastid genome were identified by tblastn [32] using as queries protein sequences encoded by the previously sequenced plastid genome of the eustigmatophyte *T. minutus*. Two and three such scaffolds were found in the *Vischeria* sp. and the *Monodopsis* sp. assemblies, respectively. Assuming the common presence of inverted repeats in plastomes and using the original sequencing reads to carry out a targeted reassembly, we obtained complete circular-mapping sequences of both plastid genomes.

Both assemblies were validated by the visual inspection of reads mapped onto the assembled consensus. To this end, Illumina genomic reads for *Vischeria* sp. CAUP Q 202 and *Monodopsis* sp. MarTras21 were subjected to trimming and quality filtering using CLC Genomics Workbench v. 8.0.3 (CLC Inc, Aarhus, Denmark) with the following settings: regions with Phred quality less than 20 were trimmed, no more than one N was allowed in the remaining sequence, then TruSeq adapter trimming and a minimum length threshold of 75 nt were applied. Filtered reads were mapped to the assembled plastid genome sequences using Bowtie2 v. 2.2.5 [33] with ‘--end-to-end’ and ‘--very-sensitive’ options. Visual inspection of the resulting read mappings in Integrative Genomics Viewer [34] validated both assemblies and led to a correction of a few indel errors in homopolymeric tracks.

### 2.2. Annotation of the *Vischeria* sp. CAUP Q 202 and *Monodopsis* sp. MarTras21 plastid genomes

An initial annotation of the two new plastome sequences was obtained using MFannot (http://megasun.bch.umontreal.ca/cgi-bin/mfannot/mfannotInterface.pl). Prediction of individual genes was checked by comparison to homologous sequences (primarily from other eustigmatophytes), which led us to revise the definition of the actual initiation codons of some of the genes. 5′ and 3′ ends of genes for non-coding RNAs (rRNAs and tRNAs) were likewise checked and adjusted by comparison to orthologous genes from other eustigmatophytes. Predicted intergenic regions were translated in all six frames and the conceptual translations were used as queries in blastp searches [32] against the non-redundant (nr) protein sequence database at the National Center for Biotechnology Information (NCBI; http://blast.ncbi.nlm.nih.gov/Blast.cgi). In parallel, the possible presence of protein-coding genes known to reside in previously sequenced eustigmatophyte plastid genomes, but not predicted by MFannot in the two newly sequenced genomes, was tested by tblastn searches against the respective plastid genome assemblies using the respective protein sequences from other eustigmatophytes. This led to the identification of some short genes missed by MFannot. A previously missed *ssrA* gene was identified in all eustigmatophyte plastid genomes thanks to the comparison of the plastid gene repertoire in different ochrophytes; its borders were delimited according to a published annotation of the gene in diatom plastomes. We also identified and incorporated into the annotation an intron-interrupted leu-tRNA gene that is conserved in eustigmatophyte plastomes [12] yet was missed by MFannot. Circular genome maps were generated with OGDraw v.1.2 [35]. A list of genes identified in plastomes of *Vischeria* sp. CAUP Q 202, *Monodopsis* sp. MarTras21 and selected other eustigmatophytes and ochrophytes is provided in the electronic supplementary material, table S1.

### 2.3. Identification of *ebo* gene homologues and analyses of bacterial *ebo* operons

Protein sequences encoded by the six genes of the novel *ebo* operon from *Vischeria* (for the definition of the operon, see §3.2) were used as queries for blastp of the NCBI nr protein sequence database. A total of 100 best hits for each query were downloaded and arrayed according to their taxonomic provenance, yielding 148 different species or strains as the main set for subsequent analyses. For each taxon in the list, the presence of homologues (orthologues) of all six *ebo* genes was checked. In cases where these homologues were not present in the initial set, we carried out dedicated blastp or tblastn searches against the genome of the respective species or evaluated by blastp or phylogenetic analyses the genes neighbouring in the genome the initially identified *ebo* genes. In several cases, some *ebo* genes were not represented by protein sequence records owing to annotation errors. Some *ebo* gene sequences proved to be partial owing to incomplete genome assembly or interrupted owing to frame-shifts. PSI-blast searches [32] were used to check those organisms for which some *ebo* genes could not be identified by the approach described above, but their genuine absence was confirmed in most cases. The relative genomic position of the *ebo* genes identified for each species (strain) was established using an in-house Python program. The electronic supplementary material, table S2, provides a list of the 148 taxa analysed, together with sequence identifiers of their Ebo proteins and the spatial arrangement of *ebo* genes in their genomes (i.e. the architecture of their *ebo* operons).

A broader analysis was carried out to investigate the origin of *eboF* homologues in eukaryotes other than eustigmatophytes. To assemble a maximally comprehensive set of eukaryotic *eboF* sequences, we searched by blastp or tblastn not only sequence databases at NCBI, but also two extensive resources of assembled transcriptomes of diverse microbial eukaryotes and plants, the Marine Microbial Eukaryote Transcriptome Sequencing Project (MMETSP; http://data.imicrobe.us/project/view/104 [36]) and the 1000 Plants project (OneKP, https://sites.google.com/a/ualberta.ca/onekp/). In addition, *eboF* homologues were also identified in the transcriptomes of *Phaeothamnion confervicola* and *Schizocladia ischiensis* (http://www.research.kobe-u.ac.jp/rcis-ku-macc/e.p.folder/e.i.folder/download.html [37]). Deduced sufficiently complete protein sequences of obvious *eboF* homologues were kept for further analysis. To detect possible closest bacterial relatives of the eukaryotic *eboF* genes, we searched the NCBI nr protein database with blastp using as a query a representative EboF sequence for each main eukaryotic group. A total of 100 best hits for each query were compared to the set of EboF homologues from the 148 bacterial species (strains) selected in the eustigmatophyte-centred analysis described above (electronic supplementary material, table S2) and non-redundant sequences were retained for a subsequent phylogenetic analysis combining all (eukaryotic and bacterial) EboF sequences thus identified (see the electronic supplementary material, table S3 for the complete list).

### 2.4. Phylogenetic analyses

All phylogenetic analyses reported in this study were based on the alignments of protein sequences built using MAFFT v7 [38] and further processed by removing unreliably aligned or too divergent regions using the GBLOCKS 0.91b program http://molevol.cmima.csic.es/castresana/Gblocks_server.html [39]) with the settings keeping the maximal number of positions in the final alignment. The alignments were analysed using the maximum-likelihood (ML) method as implemented in RAxML-HPC BlackBox (7.3.2) [40] at the CIPRES Portal (http://www.phylo.org/sub_sections/ portal/, Cyberinfrastructure for Phylogenetic Research, San Diego Supercomputing Center [41]). The LG4X substitution model [42] was used for analyses of individual protein families, except for the analysis of the large dataset covering the whole sugar phosphate cycle superfamily (see §3.4.2), which was analysed using the less computationally demanding LG model. The rapid bootstrapping algorithm (with the optimal number of bootstrap replicates chosen by the program) was employed to assess the robustness of the tree topologies. A multigene phylogenetic analysis was carried out to improve the resolution of the analysis. Individual Ebo proteins from eustigmatophytes, Bacteroidetes, Cyanobacteria and *Leptospira* spp. were aligned (in the rare cases with two paralogues of some *ebo* genes, picking only the one integrated in the operon or behaving more consistently with the *ebo* genes from the same species in single-gene trees) and concatenated using FASconCAT [43]. An ML inference was done as above, but employing the general time reversible (GTR) model, empirical base frequencies, and considering individual Ebo proteins as separate partitions. All phylogenetic trees were displayed and adjusted using iTOL (http://itol.embl.de/; [44]), with the final graphical processing done using Inkscape 0.91 (Free Software Foundation Inc., Boston, USA). Details specific for individual phylogenetic analyses are described below and in the legends to respective figures.

### 2.5. Analyses of the identity of Ebo proteins

Functional annotation of *ebo* genes was primarily attempted by the inspection of sufficiently close homologues retrieved in searches against the NCBI nr protein database with representative Ebo proteins. In addition to blastp, PSI-blast was used for some Ebo proteins using a conservative inclusion E-value threshold of 1 × 10^−5^. The Kyoto Encyclopedia of Genes and Genomes (KEGG) database [45] was also searched with blastp (http://www.genome.jp/tools/blast/) to find possible Ebo protein homologues with experimentally validated biochemical function. Representative Ebo proteins were additionally compared to collections of profile Hidden Markov Models (HMMs) in the Pfam 30.0 (http://pfam.xfam.org/ [46]) and the SUPERFAMILY 1.75 (http://supfam.org/SUPERFAMILY/ [47]) databases. In cases where Pfam and/or SUPERFAMILY searches did not retrieve significantly similar hits (EboA, EboE), we used HHpred (https://toolkit.tuebingen.mpg.de/hhpred [48]) to identify possible remote homologues using the more sensitive HMM–HMM comparison. HHalign (https://toolkit.tuebingen.mpg.de/hhalign [48]) was used for testing possible homology between EboA and EboG (see §3.2) by direct comparison of multiple alignments of EboA and EboG sequences (the alignments were built with MAFFT using sequences collected from the NCBI nr protein database with PSI-blast).

The relationship of EboB and EboC proteins to other members of broader protein superfamilies they proved to belong to was investigated using the cluster analysis implemented in the CLANS (CLuster ANalysis of Sequences) program (https://toolkit.tuebingen.mpg.de/clans [49]). For EboC, the procedure followed a recently reported analysis of the UbiA superfamily [50], which did not consider EboC proteins. Representative members of previously defined UbiA superfamily subgroups (listed in [50]) were used as queries for blastp searches against the NCBI nr database filtered for 70% maximum sequence identity (nr70). Up to 100 best hits (satisfying the E-value threshold of 1 × 10^−5^) were taken for each query. All sequences were combined with EboC homologues (electronic supplementary material, table S2) and the cluster analysis was carried out. For EboB, seed alignment sequences of all 14 families in the Amidohydrolase clan (CL0034) in the Pfam database were taken, combined with eustigmatophyte and bacterial EboB sequences (electronic supplementary material, table S2), and subjected to a cluster analysis as described above.

EboA proteins, which could not be assigned to any known conserved protein domain or family, were investigated for the possible presence of transmembrane regions using TMHMM Server v. 2 (http://www.cbs.dtu.dk/services/TMHMM/) and of N-terminal signal peptides using the SignalP 4.1 Server (http://www.cbs.dtu.dk/services/SignalP/). For the sugar phosphate cyclase (SPC) superfamily, to which EboD belongs (§3.4.2), an extended phylogenetic analysis was performed. EboD homologues collected by us in the previous step (electronic supplementary material table S2) were combined with other members of the superfamily from the same species identified by blastp and with sequences used in two previously published phylogenetic analyses of the superfamily [51,52]. Redundant sequences were removed, resulting in a set of 584 sequences that were aligned and submitted to phylogenetic inference as described above. The position of EboF within the Pfam family PF01663 (see §3.4.3) was checked by aligning a representative set of EboF sequences to the seed alignment of the family as available in the Pfam database (http://pfam.xfam.org/family/PF01663) using the ‘add’ function of MAFFT (http://mafft.cbrc.jp/alignment/server/add_sequences.html); the alignment was processed and a phylogenetic tree was inferred as generally described above.

## 3. Results and discussion

### 3.1. Basic features of the plastid genomes of *Vischeria* sp. CAUP Q 202 and *Monodopsis* sp. MarTras21

**Figure 2.**
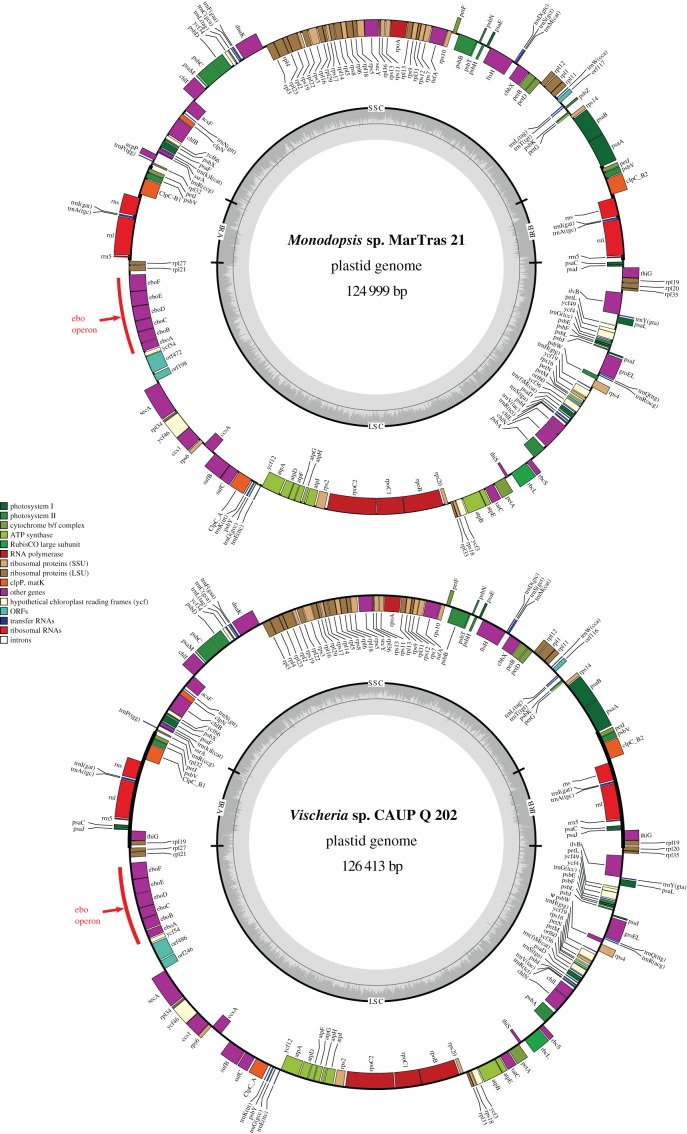
Gene maps of the newly sequenced plastid genomes of *Vischeria* sp. CAUP Q 202 and *Monodopsis* sp. MarTras21. Genes are shown as blocks facing inside if transcribed in the clockwise direction or facing outside if transcribed in the counter-clockwise direction. The assignment of the genes into different functional categories is indicated by their different colours. The plot in the inner circle shows the GC content, with the thin grey line marking 50%. Red arrows point to the novel six-gene cluster acquired by HGT. Note the *ebo* operon (*eboA* to *eboF* genes) delimited in both genomes with the red bar.

Both newly sequenced plastid genomes are circular-mapping molecules with typical inverted repeats (IRs) separating the short and the long single-copy regions (figure 2). The size of the genomes and the number, repertoire and order of genes are highly similar to those of the previously sequenced plastid genomes of other eustigmatophytes, especially *Nannochloropsis* and *Microchloropsis* species (table 1; electronic supplementary material, table S1; and data not shown). Most genes could be identified as orthologues of common plastid genes except for four ORFs conserved in eustigmatophyte plastomes but lacking discernible homologues in other organisms. In addition, six extra genes were identified in the analysed plastomes as detailed below. All previous reports on eustigmatophyte plastomes, including our own study [3,8,9,12], missed the presence of the *ssrA* gene specifying transfer-messenger RNA (tmRNA). The *ssrA* gene is situated in a previously unannotated region between two tRNA genes and this genomic position is shared with some other ochrophytes. We additionally identified unannotated *ssrA* genes in plastomes from phaeophytes, whereas the chrysophyte *Ochromonas* sp. CCMP1393 and the xanthophyte *Vaucheria litorea* appear to lack this gene (or it has diverged beyond recognition in these taxa; electronic supplementary material, table S1).

**Table 1.** Basic features of plastid genomes of eustigmatophyte algae. For simplicity, only one species is included for each of the genera *Nannochloropsis* (i.e. *N. oculata* CCMP525) and *Microchloropsis* (i.e. *M. salina* CCMP1776).

	*Vischeria* sp. CAUP Q 202	*Monodopsis* sp. MarTras21	*N. oculata* CCMP525	*M. salina* CCMP1776	*T. minutus* CCALA 838
size (bp)	126 413	124 999	117 463	114 821	120 091
inverted repeat (bp)	9795	7870	7476	5129	9412/9411
large single-copy region (bp)	62 270	63 612	57 287	56 872	56 060
small single-copy region (bp)	44 553	45 647	45 227	47 691	45 210
total GC content (%)	32.49	32.76	33.36	33.09	33.98
gene content (total)	165	168	163	160	163
identified common plastid protein-coding genes	124	127	126	126	128
non-typical conserved genes	6	6	0	0	0
unknown ORFs	4	4	4	4	4
pseudogenes	1	0	0	0	0
rRNA genes	3	3	3	3	3
tRNA genes	26	26	26	25	28
other non-coding RNA genes	1	1	1	1	1
number of genes in inverted repeat	12	8	8	5	12

Except for the varying size of the inverted repeats (i.e. a different copy number of some genes; table 1), the differences in the gene repertoire of individual eustigmatophytes primarily result from differential gene loss. Previously, the genes *acpP* (coding for acyl carrier protein), *lysR* (i.e. *rbcR*; encoding a transcription regulator of the RuBisCO operon) and *tsf* (encoding translation elongation factor Ts) were identified in the plastome of *T. minutus*, but were found missing from plastomes of *Nannochloropsis* (incl. *Microchloropsis*) [12]. Our present study documents the absence of *lysR* and *tsf* also from the *Vischeria* and *Monodopsis* plastomes, suggesting their loss early in the evolution of Eustigmatales. However, the presence of an *acpP* gene in the *Monodopsis* plastome, but not that of *Vischeria*, indicates that there were at least two independent losses of *acpP* in the eustigmatophyte evolution (since *Monodopsis* is specifically related to *Nannochloropsis*/*Microchloropsis*; figure 1). The most notable losses impacting terminal eustigmatophyte taxa concern the *Vischeria* plastome. Specifically, it proved to lack the *psbZ* gene encoding a subunit of photosystem II (electronic supplementary material, table S1). The region corresponding to another photosystem II gene, *psbW*, is present in the *Vischeria* plastome and looks mostly intact, but a single-nucleotide deletion (confirmed with inspection of reads mapped to the region) has caused a frame-shift mutation in the coding sequence that probably makes the gene non-functional (hence it is considered a pseudogene in table 1 and electronic supplementary material, table S1). Neither of the plastid gene losses mentioned above are unique to eustigmatophytes, as the respective genes are also missing from some other ochrophyte plastomes we analysed (electronic supplementary material, table S1). Future analyses of nuclear genomes will reveal whether these are genuine losses or whether nuclear genes exist to encode homologous or functionally analogous plastid-targeted proteins.

The most striking finding yielded by our analyses is that the eustigmatophyte plastomes have been sculpted not only by gene losses, but also by gene gains. In the previously sequenced plastomes of *T. minutus*, *Nannochloropsis* spp. and *Microchloropsis* spp., the genes *ycf54* and *rpl21* are direct neighbours [3,8,9,12], but the plastomes of *Vischeria* sp. CAUP Q 202 and *Monodopsis* sp. MarTras21 surprisingly proved to exhibit a cluster of six putative genes inserted between *ycf54* and *rpl21* (figure 2). Blast searches against the non-redundant protein and nucleotide sequence databases at the NCBI revealed that neither of these genes has a homologue in any of the plastid genomes sequenced so far, but homologues could be readily identified in various bacteria. This suggested that the whole cluster was most probably acquired by an ancestor of *Vischeria* and *Monodopsis* by HGT from a bacterial source. In the rest of this report, we provide a detailed analysis of this novel gene cluster in order to elucidate its evolutionary origin and biological significance.

### 3.2. A novel six-gene operon shared by plastid genomes of *Vischeria* sp. CAUP Q 202 and *Monodopsis* sp. MarTras21 and many bacteria

**Figure 3.**
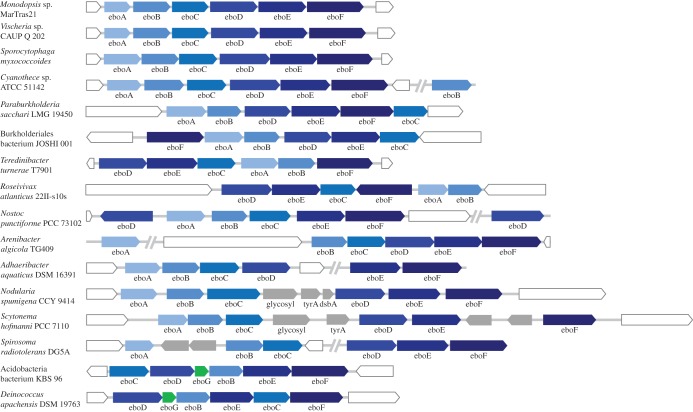
The *ebo* operon, a novel six-gene operon shared between the plastid genomes of some eustigmatophytes and bacteria. The figure shows examples of various arrangements of the *ebo* operon. Genes (or predicted ORFs) are displayed as boxes with the pointed side indicating the 3' end of the coding sequence (i.e. the direction of transcription). Accession numbers of protein sequences encoded by the *ebo* genes included in the figure are provided in the electronic supplementary material, table S2. Boxes in white correspond to genes that flank clusters of *ebo* genes in the genomes but are not considered to be a part of the *ebo* operon itself. Boxes in grey correspond to non-*ebo* genes presumably inserted into the original *ebo* operon. Double slashes indicate intervening genomic sequences of a varying length (sometimes including a break in the genome sequence assembly). Note that in some species an extra copy of one of the *ebo* genes exists in the genome, detached from the *ebo* operon as such.

To illuminate the origin and function of this gene cluster, we carried out a more detailed investigation of the homologous genes in bacteria. Using blastp, we retrieved and analysed 100 best hits in the NCBI nr protein database for each of the six proteins encoded by the cluster in the *Vischeria* plastome (we did not repeat the same procedure for *Monodopsis*, as its sequences are highly similar to those from *Vischeria*). All these hits came from various bacterial species representing seven different phyla, with the phylum Bacteroidetes being most represented followed by the phylum Cyanobacteria. The hits for the six different queries often came from the same bacterial species and closer inspection revealed that they are, in most cases, encoded by genes that are physically close to each other in the genome of the respective bacterium. This suggested the existence of gene clusters similar to that in the *Vischeria* and *Monodopsis* plastid genomes. Therefore, we systematically looked for homologues of all six genes in every bacterial species present at least once among the best 100 hits for any of the original queries (altogether 148 species/strains) and defined their relative position within the genome (details provided in the electronic supplementary material, table S2). Crucially, this analysis confirmed that, in most cases, the bacterial homologues of all six eustigmatophyte plastid genes are arranged in clusters, although the actual order of the genes and even the very occurrence of some of the genes in the cluster varied (figure 3; electronic supplementary material, table S2; and see below). An exception was found in representatives of the phylum Planctomycetes, where no clustering was observed in the three species represented in our analysis.

Such generally consistent physical clustering of homologous genes in bacterial genomes strongly suggests that the genes constitute a functional module and most probably a single transcription unit, i.e. an operon. As discussed in detail below, it seems that no gene of the novel operon analysed here has been experimentally characterized in any species and, hence, no formal name has been proposed for any of the genes. Furthermore, the operon is not listed in DoBISCUIT, a manually curated and regularly updated database of secondary metabolite biosynthetic gene clusters (http://www.bio.nite.go.jp/pks/ [53]). To facilitate communication about the new operon, we denote it ‘*ebo*’, standing for the ‘eustigmatophyte and bacterial operon’. The six genes conserved between eustigmatophytes and bacteria are consequently labelled *eboA* to *eboF*, following the order in the originally found, i.e. eustigmatophyte, version of the operon (figure 3). The corresponding proteins encoded by the *ebo* genes are then named EboA to EboF.

Having established the existence of the *ebo* operon, we further investigated its variation in bacteria. We restricted the analysis to the 148 taxa presented in the electronic supplementary material, table S2, although blastp and PSI-blast searches revealed that *ebo* gene homologues exist in many other bacteria (data not shown). It is likely that we have missed a part of the variation concerning the *ebo* operon structure, but our restricted sampling strategy did provide numerous insights. Most importantly, many bacteria exhibit exactly the same arrangement of the *ebo* genes as the two eustigmatophytes (figure 3; electronic supplementary material, table S2). This indicates that the whole cluster could have been directly transferred *en bloc* from a bacterial source into a eustigmatophyte plastome. There is a clear phylogenetic pattern in the distribution of this *ebo* operon architecture (denoted ABCDEF in the electronic supplementary material, table S2) in bacteria, as it is restricted to members of the phyla Bacteroidetes, Cyanobacteria and the genus *Leptospira* (phylum Spirochaetes). Hence, the putative donor of the eustigmatophyte *ebo* operon most probably belonged to one of these groups (this issue is addressed by phylogenetic analyses in the subsequent section).

Nevertheless, many taxa analysed here depart in many different ways from the ABCDEF-type *ebo* operon structure. The few deviations seen in some Bacteroidetes and Cyanobacteria members concern terminal phylogenetic branches, hence the ABCDEF-type *ebo* operon is apparently the ancestral form in both phyla. Alternative arrangements of the six *ebo* genes are typical for other bacterial phyla (except spirochaetes of the genus *Leptospira* that acquired their ABCDEF-type *ebo* operon via HGT from Bacteroidetes, see §3.3), including ABDEFC, FABDEC or DECABF (figure 3; electronic supplementary material, table S2). In some species, all six genes are physically clustered in the genome, but cannot be considered a single operon because of a reversed orientation of one of the genes with respect to the others (figure 3). In other species, the operon is disrupted by physical separation of the *ebo* genes, as is the case of the genus *Arenibacter* and in *Adhaeribacter aquaticus* (both Bacteroidetes) having separated the *eboA* gene and the *eboE–eboF* gene pair, respectively, from the remaining *ebo* genes. We also encountered cases where an extra copy of an *ebo* gene can be found in a locus distant from the *ebo* operon itself, as in the case of an extra *eboB* copy in *Cyanothece* sp. ATCC 51142 (Cyanobacteria). Other modifications of the *ebo* operon architecture have originated from insertions of non-*ebo* genes into the operon. This has been documented in several Cyanobacteria species sharing two or three conserved genes inserted between *eboC* and *eboD*: a putative glycosyltransferase, prephenate dehydrogenase (*tyrA*) and dithiol–disulfide isomerase (*dsbA*; see *Nodularia spumigena* and *Scytonema hoffmanni* in figure 3). In other cases, the extra genes inserted into the *ebo* operon code for uncharacterized or hypothetical proteins. An analysis of these genes is beyond the scope of this study.

Although there is a clear tendency for co-occurrence of all six *ebo* genes in a genome, we did find cases where no orthologue of a particular *ebo* gene could be identified in the genome. *Leeuwenhoekiella* sp. MAR_2009_132 (Bacteroidetes) has an *ebo* operon without an *eboD* gene (ABCEF) and no *eboD* orthologue could be identified in the available draft genome assembly. This may be due to a recent deletion of the *eboD* gene, but genome assembly artefacts cannot be excluded. Two of the three members of the phylum Planctomycetes analysed here also lack *eboD* orthologues (electronic supplementary material, table S2), yet their genome assemblies are also of the draft quality. A sequencing gap clearly explains the absence of an *eboC* gene in *Limnoraphis robusta* (Cyanobacteria), as an *eboA–eboB* gene pair is found at the very end of one of the contigs, whereas a cluster of *eboD*, *eboE* and *eboF* genes is located at the very beginning of another contig (electronic supplementary material, table S2). An extreme case of *ebo* gene loss is the cyanobacterium *Nostoc* sp. PCC 7524, possessing only an *eboD* gene. Given the occurrence of a complete *ebo* operon in a number of closely related cyanobacteria, the absence of most *ebo* genes in *Nostoc* sp. PCC 7524 is probably a result of a recent loss.

Interestingly, a group of species proved to exhibit a variant of an *ebo* operon consistently lacking the *eboA* gene (encoding a short moderately conserved protein; see §3.4.4). The species that belong to this category include Acidobacteriaceae bacterium KBS 96 (Acidobacteria), *Thermithiobacillus tepidarius* (Proteobacteria), *Herpetosiphon* spp. (Chloroflexi) and members of Deinococcales (*Deinococcus* spp. and *Truepera radiovictrix*). No *eboA* homologues could be identified in these taxa even when an exhaustive PSI-blast search was used. Instead, all these species possess a novel conserved uncharacterized gene, hereafter provisionally denoted as *eboG*, that clusters with the other *ebo* genes in an alternative version of the *ebo* operon (figure 3; electronic supplementary material, table S2). The predicted EboG proteins are short and not well conserved, resembling in this sense EboA. We tested the possibility that EboG and EboA are, in fact, remotely homologous using HHalign (employing the highly sensitive comparison of profile HMMs), but the result was negative. Our extended search confirmed the presence of *eboG* homologues in many other bacterial species and preliminary results indicate that it is typically associated with the remaining *ebo* genes (data not shown). Thus, there might be at least two main *ebo* operon variants widespread in bacteria, distinguished by the mutually exclusive presence of *eboA* or *eboG.* However, unclear biochemical function of both EboA and EboG (see §3.4.4) makes it impossible to deduce whether the two *ebo* operon variants govern biosynthesis of different final products or whether they impact other functional aspects of the operon, such as its regulation.

### 3.3. The evolutionary origin of the eustigmatophyte *ebo* operon

The analyses presented above establish the presence of the *ebo* operon in the *Vischeria* and *Monodopsis* plastid genomes as an obvious case of horizontal gene (operon) transfer from a bacterium. The salient remaining evolutionary questions are what were the recipient and the donor of this transfer.

As to the first question, correlating the occurrence of the operon with the eustigmatophyte phylogeny [10,14] needs to be considered. *Vischeria* and *Monodopsis* both belong to the order Eustigmatales, whereas the second major eustigmatophyte group, the clade Goniochloridales represented by the plastome sequence from *T. minutus*, lacks the *ebo* operon (figure 1). The most parsimonious scenario thus is that the *ebo* operon was acquired only after the split of Goniochloridales and Eustigmatales. Within the latter group, *Vischeria* and *Monodopsis* represent two of the three principal lineages, specifically the Eustigmataceae group and the family Monodopsidaceae [10]. Thus, the *ebo* operon must have been acquired before the split of these two lineages. However, so far we lack plastid genome data from the third main lineage of the order Eustigmatales, the so-called *Pseudellipsoidion* group. Moreover, the branching order of the three main Eustigmatales lineages has not been robustly resolved yet [10]. Hence, it is presently unclear whether the acquisition of the operon preceded the radiation of the whole Eustigmatales group or whether it occurred within a particular Eustigmatales lineage. Importantly, the absence of the *ebo* operon from all sequenced plastid genomes of the genera *Nannochloropsis* and *Microchloropsis*, together constituting a sister group to the *Monodopsis* lineage [7], implies a secondary loss of the operon from that group (figure 1).

**Figure 4.**
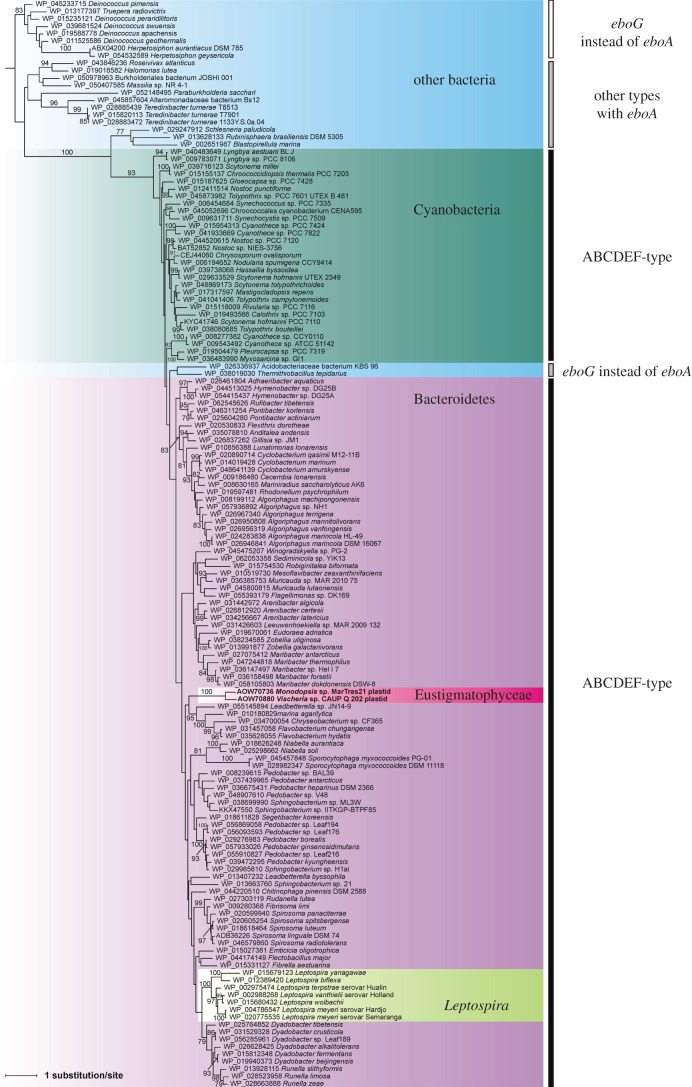
Phylogenetic analysis of EboC protein sequences. The ML trees were inferred from an alignment of 224 amino acid positions using RAxML and the LG4X+Γ substitution model. Bootstrap support values are shown at branches when more than 75%. Five groups of species are distinguished by a different background: Bacteroidetes, violet; Cyanobacteria, blue-green; Eustigmatophyceae, red; *Leptospira*, light green; other bacteria, blue. Taxa are divided into three conveniently defined groups according to the type of *ebo* operon they possess (see the vertical bars on the right side). Note that some species assigned in the figure to the ABCDEF-type of the operon secondarily deviate from this presumed ancestral form for the given major taxa (see §3.2).

To answer the question on the nature of the putative bacterial donor, we first constructed phylogenetic trees for each of the six encoded proteins. Specifically, we analysed alignments of the two eustigmatophyte sequences along with homologues from the bacterial species (strains) selected as described above (electronic supplementary material, table S2). The trees were generally poorly resolved, perhaps owing to the limited length of the Ebo proteins and, hence, a limited phylogenetic signal in their sequences (figure 4 and electronic supplementary material, figure S1). In all six cases, the two eustigmatophyte sequences clustered together with maximal bootstrap support, confirming that the *ebo* operon was acquired only once in eustigmatophytes, but the position of the eustigmatophyte branch varied between the trees. In three trees (for EboA, EboC and EboE) the eustigmatophyte branch was nested among sequences from the phylum Bacteroidetes, and in one tree (EboF) it was sister to Bacteroidetes sequences, but with low bootstrap support values in all cases. In the remaining two trees (EboB and EboD), the eustigmatophyte branch was found sister to Cyanobacteria, but again with low bootstrap support. Such conflicting or unclear results are commonly observed in phylogenetic analyses concerning ancient HGT events, and, indeed, they rarely point with confidence to a particular bacterial taxon as a source of bacteria-derived eukaryotic genes (e.g. [54,55]).

Nevertheless, the phylogenetic trees for individual Ebo proteins did provide additional insights into the evolutionary history of the operon. The EboB tree shows with maximal bootstrap support a bipartition separating taxa with *eboA* on the one side and *eboG* on the other side (electronic supplementary material, figure S1*b*). This supports the notion of the existence of two principal variants of the operon. The EboC phylogeny (figure 4) suggests a more complex scenario. The tree topology is generally consistent with separating taxa according to the presence of *eboA* or *eboG*, and further according to the *ebo* operon architecture ancestral for the respective taxa—the ABCDEF-type versus other arrangements. However, two taxa, Acidobacteriaceae bacterium KBS 96 and *Thermithiobacillus tepidarius*, disturb this simple picture by being nested (with bootstrap support of 93%) within the ABCDEF-type group. The most probable explanation is replacement of the original *eboC* gene in these taxa with a homologue from a bacterium representing the ABCDEF-type group. Indeed, separation of taxa (ancestrally) with the ABCDEF-type *ebo* operon from other operon arrangements (including Acidobacteriaceae bacterium KBS 96 and *Thermithiobacillus tepidarius*) is retained (with moderate bootstrap support of 78%) in the EboE tree (electronic supplementary material, figure S1*d*). EboA and EboF trees (electronic supplementary material, figure S1*a,e*) are topologically consistent with this split as well, although without good statistical support, while the EboD tree has a mixed topology in this respect, yet the discrepancies have low statistical support (electronic supplementary material, figure S1*c*). Altogether, these phylogenetic analyses suggest that the *ebo* operons in eustigmatophytes, Bacteroidetes, Cyanobacteria and the genus *Leptospira* are all derived from a common ABCDEF-type operon architecture and are evolutionarily separated from *ebo* operons from other taxa.

**Figure 5.**
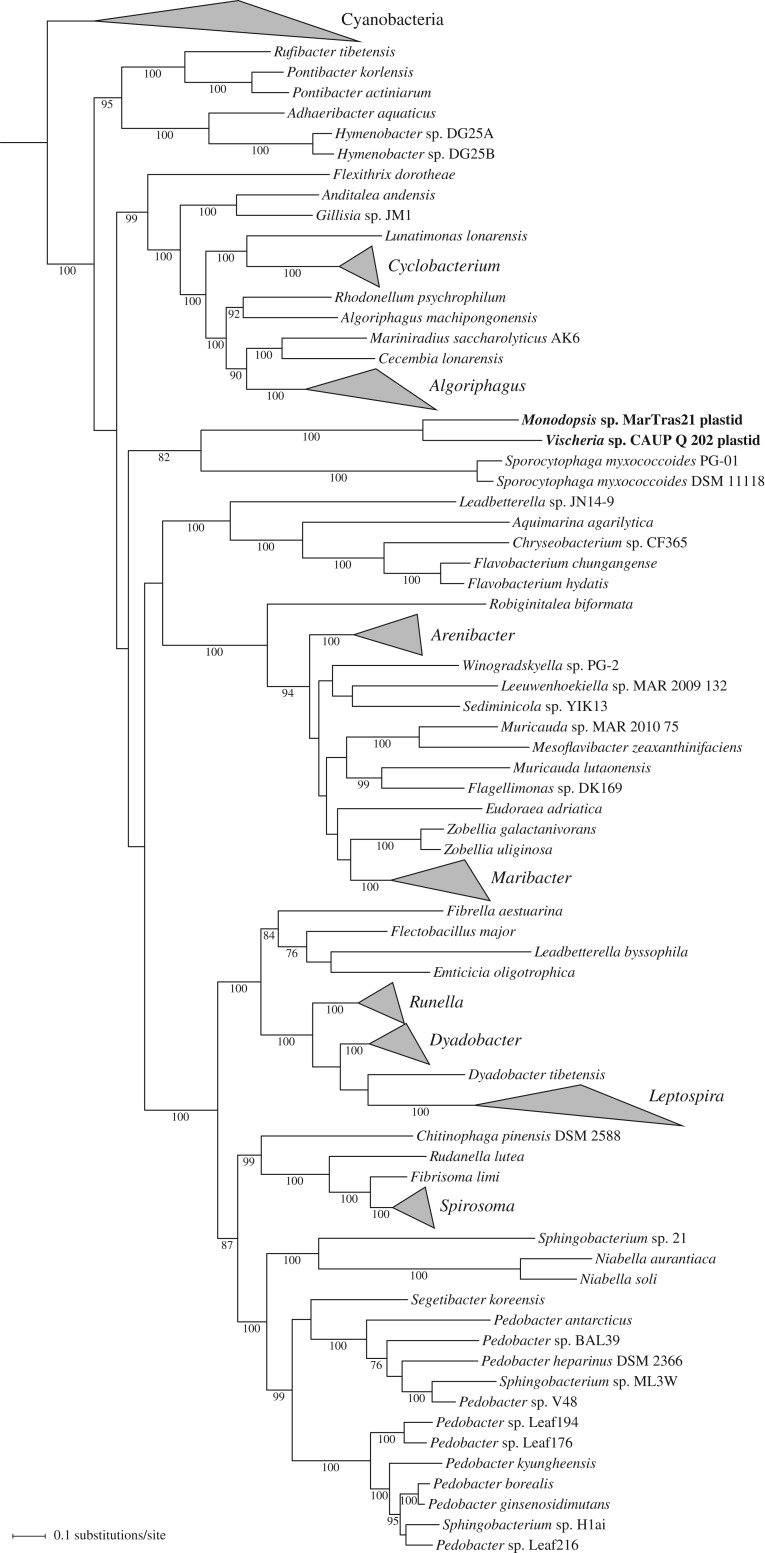
The origin of the novel operon in the eustigmatophyte plastid genomes can be traced to a bacterium from the phylum Bacteroidetes. The phylogenetic tree displayed was inferred with the ML method and the GTR+Γ substitution model from a concatenated alignment of protein sequences of all six *ebo* genes (1838 aligned amino acids). Bootstrap support values are shown at branches when more than 75%. The tree is arbitrarily rooted on a maximally supported branch separating Cyanobacteria and other taxa included in the analysis. For simplicity, monophyletic groups comprising exclusively representatives of the same genus were collapsed and are shown as triangles, with the two side lengths proportional to the distances to closest and furthest leaves. Note that sequences from the eustigmatophytes *Vischeria* sp. CAUP Q 202 and *Monodopsis* sp. MarTras21 branch among members of the phylum Bacteroidetes, possibly specifically related to the *ebo* operon from *Sporocytophaga myxococcoides*. Note also that the cluster of sequences from the genus *Leptospira* (phylum Spirochaetes) is firmly nested among Bacteroidetes, indicating an inter-phylum HGT event.

To improve the resolution of phylogenetic analysis, we inferred a tree from a concatenated alignment of all six Ebo proteins, restricting the analysis only to the aforementioned taxa with the ABCDEF-type operon. The resulting tree (figure 5), indeed, shows much higher bootstrap support values for most branches and further clarifies the origin of the *ebo* operon in eustigmatophytes. The tree clearly supports the scenario suggested by some of the single-gene trees that the eustigmatophyte operon is derived from within the phylum Bacteroidetes. The closest known relative of the eustigmatophyte *ebo* operon may be that of *Sporocytophaga myxococcoides*, with this relationship being supported by a moderate bootstrap value of 82%. Future genome sampling may pinpoint the original source of the eustigmatophyte operon with a higher precision. Notably, our phylogenetic analyses provide strong support for another horizontal transfer of the *ebo* operon from a member of Bacteroidetes (possibly a species of the genus *Dyadobacter*), specifically to a lineage of spirochaetes representing a deeply diverged monophyletic subgroup of the genus *Leptospira* [56].

The Bacteroidetes phylum has been demonstrated as an important source of bacterial genes acquired by different eukaryotes [57], including algae (e.g. [58]), and bacteria of the Bacteroidetes group are known to physically interact with various algae (e.g. [59–61]). Bacteroidetes’ representatives occur in diverse habitats, including freshwater and soil, and *S. myxococcoides*, the putative closest relative of the donor of the eustigmatophyte *ebo* operon, is a cellulose-degrading soil bacterium [62]. Physical association of the putative donor of the *ebo* operon with eustigmatophytes, which are mostly freshwater or soil algae [63], is thus conceivable. It is interesting to note in this context that a case for an acquisition of Bacteroidetes-derived genes by plastid genomes has already been raised before. Moszczynski *et al*. [64] suggested that the plastomes of the dinoflagellates *Ceratium horridum* and *Pyrocystis lunula* contain genes (homologous to genes in plastomes of some other algal groups but not dinoflagellates so far) that were gained by HGT from a Bacteroidetes donor. However, as already pointed out by Dorrell & Howe [65], this may in fact be a misidentified bacterial contamination of the plastid genome data from these two species. Indeed, the respective DNA sequences (GenBank accession numbers AF490364 and AF490367) are both incomplete and do not bear any obvious signatures of belonging to a dinoflagellate plastid genome (2016, our unpublished observation).

### 3.4. Searching for the function of the *ebo* operon genes

Although the discovery of the *ebo* operon in the eustigmatophyte plastomes and its occurrence in a wide array of bacteria is intriguing, the central unanswered question concerns the actual biological role of this gene cluster. While surveying the literature and sequence databases (NCBI and KEGG), we did not encounter any close homologue of any of the Ebo proteins for which its biochemical function would have been tested experimentally. Therefore, for the moment we have to rely on *in silico* approaches to get some insights. We first discuss Ebo proteins for which more specific predictions concerning their function can be made, and conclude with the EboA protein, whose function remains elusive. Functional annotation of individual Ebo proteins predicted by our analyses is summarized in table 2.

**Table 2.** Functional annotation of Ebo proteins.

protein	predicted function	details
EboA	novel protein, no functional assignment	no homologues identified even with HHsearch
EboB	putative hydrolase (but different enzymatic activities cannot be excluded)	assigned to the PF01026 family (‘TatD-related DNase’) by Pfam search, but appears to be a separate cluster of the same clan (CL0034 – ‘Amidohydrolase’; electronic supplementary material, figure S3); ‘Metallo-dependent hydrolase’ by SUPERFAMILY
EboC	putative prenyltransferase	UbiA superfamily of intramembrane prenyltransferases [50] based on blastp and Pfam searches (PF01040); proved to comprise a separate cluster within the superfamily (figure 6)
EboD	putative SPC, sedoheptulose 7-phosphate may be the substrate	belongs to SPC superfamily [66], equal to the PF01761 family (‘3-dehydroquinate synthase’) in Pfam, but a separate branch within the superfamily putatively with a novel specificity (figure 7) rather than true DHQS
EboE	TIM-barrel protein of the xylose isomerase-like superfamily, no more specific prediction of the enzyme activity possible	Some EboE sequences marginally similar to the PF01261 family (‘Xylose isomerase-like TIM barrel’) by Pfam search; ‘Xylose isomerase-like’ by SUPERFAMILY; homologous to TIM-barrel proteins according to HHsearch
EboF	putative hydrolase specific for a phosphate moiety-containing substrate	PF01663 family (‘Type I phosphodiesterase/nucleotide pyrophosphatase’) by Pfam search; ‘Alkaline phosphatase-like’ by SUPERFAMILY; a distinct phylogenetic lineage within the PF01663 family (electronic supplementary material, figure S2)

#### 3.4.1. EboC is a novel member of the UbiA superfamily of intramembrane prenyltransferases

Many of the bacterial EboC proteins are annotated as ‘UbiA prenyltransferase’ or ‘polyprenyltransferase’ in the NCBI protein sequence database. This is consistent with the fact that querying the Pfam database with EboC proteins assigns them to the ‘UbiA prenyltransferase family’ (PF01040) with high statistical support. Most of the EboC protein length aligns to the UbiA family model in Pfam and *vice versa*, and no other additional conserved domain could be found in EboC proteins, indicating that they entirely correspond to UbiA homologues. UbiA, an enzyme (4-hydroxybenzoate polyprenyltransferase) involved in ubiquinone biosynthesis [67], is a prototype of a much broader group of proteins with a similar structure and biochemical function, recently denoted as the UbiA superfamily [50]. Members of this group are intramembrane proteins that catalyse attachment of (poly)prenyl chains or their derivatives to different substrates. In addition to ubiquinone biosynthesis mediated by UbiA itself, other metabolic pathways involving members of the UbiA superfamily include the synthesis of other membrane-bound electron carriers (menaquinone, plastoquinone), chlorophyll, haem O, vitamin E (tocopherol), glycerol diether and tetraether lipids in Archaea, and a growing number of other substances including secondary metabolites, such as the antimicrobial compound shikonin [50].

**Figure 6.**
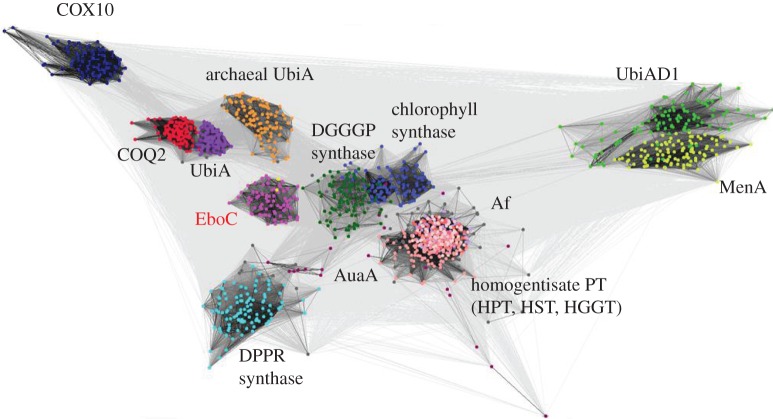
Cluster analysis of sequences of the UbiA superfamily showing that EboC belongs to a novel separate group within the superfamily. Each dot represents a sequence and each grey line shows a PSI-BLAST comparison of two sequences, with darker lines indicating higher similarity (lower E-values). The cluster of EboC homologues is coloured bright pink, with *Monodopsis* and *Vischeria* sequences highlighted in yellow. Other members of the UbiA superfamily include COQ2 (red), UbiA (magenta), UbiAD1 (green), MenA (yellow green), homogentisate prenyltransferases (HPT, HST and HGGT in different pink colours), chlorophyll synthase (blue), COX10 (dark blue), DGGGP synthase (dark green), DPPR synthase (cyan), archaeal UbiA homologues (orange), AuaA homologues (wine colour) and Af homologues (dark grey).

It has been noted previously that there are many members of the UbiA superfamily with unknown and possibly novel substrate specificity, which cannot be predicted from the protein sequence alone [50]. We investigated the relationship of EboC proteins to the previously studied members of the UbiA superfamily. Despite obvious homology, the sequence divergence within the superfamily is rather high, limiting the use of standard phylogenetic analyses. Therefore, we applied a sequence clustering method recently used for mapping the sequence space of the UbiA superfamily, yet without EboC proteins (see fig. 1 in [50]). Our analysis revealed that EboC proteins constitute a distinct sequence cluster within the UbiA superfamily (figure 6). Its substrate specificity cannot be reliably determined, but we predict that EboC proteins serve as enzymes catalysing prenylation of a yet-to-be-defined substrate. Prenyl chain donors (prenyl diphosphates) of different length are commonly synthesized in plastids of all algal groups [68], so the postulated substrate of EboC is presumably available in the eustigmatophyte plastids.

#### 3.4.2. EboD is a novel member of the sugar phosphate cyclase superfamily

EboD proteins are typically annotated as ‘3-dehydroquinate synthase’ in the NCBI protein database and Pfam recognizes them with a very low E-value as members of the ‘DHQ_synthase’ family (PF01761), so the predicted biochemical function of EboD would seem obvious. However, proteins with the actual 3-dehydroquinate synthase (DHQS) activity, also called AroB, are, in fact, members of a much broader group of enzymes catalysing similar reactions yet using different substrates and taking part in very different metabolic pathways [51,66]. DHQS is an important component of primary metabolism, catalysing one of the critical steps of the shikimate pathway leading to aromatic amino acids (specifically conversion of 3-deoxy-d-*arabino*-heptulosonate 7-phosphate into 3-dehydroquinate). However, a number of DHQS (AroB) homologues are known to catalyse different reactions in pathways producing various secondary metabolites, primarily in bacteria [51,66], but more recently also vertebrates [52]. All these enzymes are united in the SPC superfamily [66], and some of its members have attracted attention owing to their role in the biosynthesis of important bioactive compounds, including aminoglycoside antibiotics (e.g. kanamycin and neomycin), the antidiabetic drug acarbose, mycosporine-like amino acids (natural sunscreens), the anti-tumour agent cetoniacytone A, or the crop protectant validamycin A [51,66,69].

**Figure 7.**
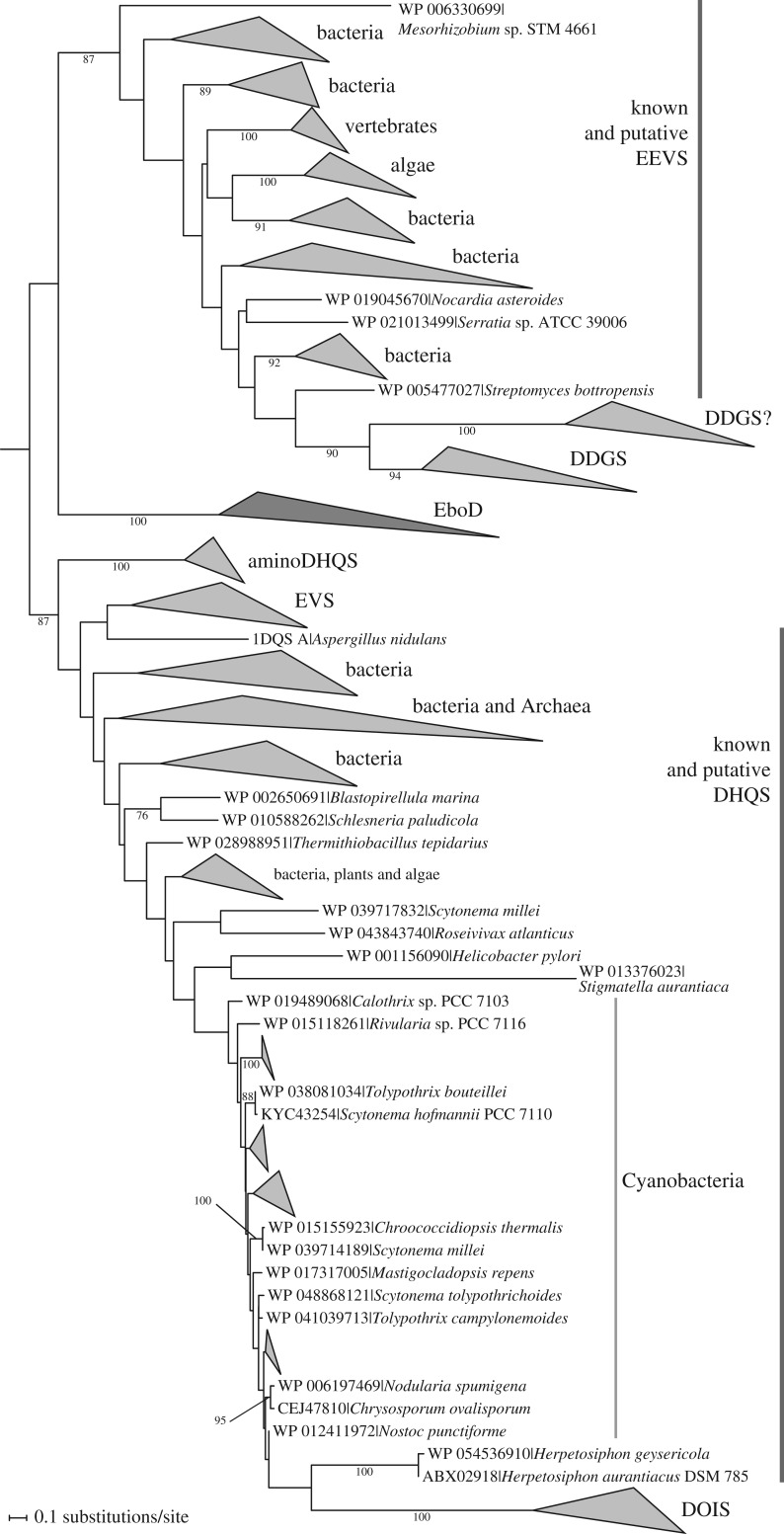
EboD represents a new lineage within the SPC superfamily. The phylogenetic tree displayed was inferred with the ML method using the LG+Γ substitution model and a protein sequence of alignment of 147 amino acids. For simplicity, most monophyletic branches of the same putative enzymatic activity with more than two members were collapsed as in figure 5, bootstrap support values are shown only when more than 75%. The tree was rooted at a position suggested by a previously published analysis using glycerol dehydrogenase (CglD) from *Escherichia coli* as an outgroup [52]. Main subgroups of the superfamily characterized by different enzymatic activities are indicated. Note that most proteins in the tree have not been characterized biochemically, so the functional assignment suggested by the tree topology has to be taken with caution (for example, the clade including confirmed EVS enzymes would apparently be considered as putative DHQS without the actual data to the contrary). AminoDHQS, aminodehydroquinate synthase; DDGS, desmethyl-4-deoxygadusol synthase; DDGS?, a clade of uncharacterized proteins related to DDGS, but distinctly different to possibly exhibit a different enzymatic activity; DHQS, 3-dehydroquinate synthase; DOIS, 2-deoxy-*scyllo*-inosose synthase; EEVS, 2-epi-5-epi-valiolone synthase; EVS, 2-epi-valiolone synthase.

To determine the identity of EboD proteins, we carried out a phylogenetic analysis of the SPC superfamily, made possible because of the sufficiently high sequence similarity of different superfamily members. In the analysis, we included representative sequences of all known enzymatically distinct members as well as a selection of sequences with undetermined biochemical specificity. The resulting phylogenetic tree (figure 7) shows EboD proteins as a new, highly supported clade within the superfamily, distinctly different from the true DHQS proteins and all other previously delineated subgroups. Hence, the annotation of many EboD proteins in databases as 3-dehydroquinate synthase is unfounded and simply an example of the limits of automatic approaches that transfer functional annotation to genes based purely on sequence similarity. We can only speculate what might be the substrate and the product of EboD-catalysed reaction, but notably, EboD is placed in a part of the SPC superfamily tree that include enzymes known to use sedoheptulose 7-phosphate as the substrate to make several different products. One of them is 2-epi-5-epi-valiolone synthase (EEVS) that converts sedoheptulose 7-phosphate to 2-epi-5-epi-valiolone, a precursor for the synthesis of acarbose, validamycin A, a natural sunscreen gadusol and a number of other compounds [52,66,69]. Another enzyme phylogenetically close to EboD is desmethyl-4-deoxygadusol synthase (DDGS) that converts sedoheptulose 7-phosphate to desmethyl-4-deoxygadusol, a precursor of mycosporine-like amino acids that also serve as protectants against excessive light and other environmental stress factors [70]. The recently characterized reaction converting sedoheptulose 7-phosphate is catalysed by 2-epi-valiolone synthase (EVS) and leads to 2-epi-valiolone, a putative precursor of hitherto uncharacterized final secondary metabolites [51].

Taking into account the phylogenetic position of EboD within the SPC superfamily and the known substrate specificity of its relatives, we posit that EboD catalyses conversion of sedoheptulose 7-phosphate into a cyclic hydrocarbon with multiple hydroxy groups, i.e. a cyclitol (similar to, if not identical with, 2-epi-5-epi-valiolone or another product mentioned above). In most organisms, the source of sedoheptulose 7-phosphate is the pentose phosphate pathway, but this substance is also an intermediate of the Calvin cycle, which in eukaryotes is located to plastids. Hence, the EboD proteins in eustigmatophytes would have an access to their postulated substrate in the plastid.

#### 3.4.3. EboF is a putative hydrolase specific for a phosphate moiety-containing substrate

EboF proteins in the NCBI database are typically annotated as ‘phosphodiesterase’ or ‘alkaline phosphatase family protein’. The former is consistent with the results of searches against the Pfam database, which assigned EboF proteins with high confidence to the family PF01663, annotated as ‘Type I phosphodiesterase/nucleotide pyrophosphatase’. The latter annotation perhaps reflects the fact that this family belongs to the Pfam clan CL0088 named ‘Alkaline phosphatase-like’. SUPERFAMILY database searches also assigned EboF proteins to the ‘Alkaline phosphatase-like’ superfamily, but not into a particular subordinate family, consistent with the absence of a solved protein structure for any close EboF homologue. We performed a phylogenetic analysis of a subset of EboF proteins with the sequences used to build the seed alignment of the PF01663 family in Pfam. The analysis revealed that EboF sequences (including five from the seed alignment) make a cluster well separated from the other sequences analysed (electronic supplementary material, figure S2), supporting the notion that EboF constitutes a separate lineage within the Type I phosphodiesterase/nucleotide pyrophosphatase family. The experimentally studied members of the family catalyse hydrolysis of phosphoanhydride or phosphodiester bonds in various substrates, including nucleotides and their derivatives, lysophospholipids or choline phosphate esters [71]. It is, therefore, conceivable that EboF catalyses hydrolysis of a phosphate moiety-containing substrate, but a more specific prediction cannot be made by *in silico* analyses alone.

#### 3.4.4. Only general functional predictions can be made for EboB and EboE, and little can be said about the EboA protein

Most EboB proteins are annotated as ‘hydrolase TatD’ in the NCBI database, consistent with our searches against the Pfam database, which assigned EboB sequences to the Pfam family PF01026 annotated as ‘TatD related DNase’. However, the E-values for this assignment were relatively high (from as high as 1 × 10^−5^ to 1 × 10^−21^ at best for different EboB proteins), with none of the sequences used to build the seed alignment of the family being particularly similar to EboB. Searching the SUPERFAMILY database assigned EboB sequences with high confidence to the metallo-dependent hydrolases superfamily, which roughly corresponds to the Amidohydrolase superfamily (i.e. clan CL0034 including the PF01026 family) defined in the Pfam database. The SUPERFAMILY search did not assign EboB to any particular family because of the E-value threshold, but the ‘TatD Mg-dependent DNase-like’ family was identified as the most similar. These observations suggest that EboB is a member of the metallo-dependent hydrolase (or amidohydrolase) superfamily, possibly affiliated to the TatD DNase family. To get a better view of the position of EboB within this broader protein grouping, we performed a cluster analysis using CLANS. It showed that EboB sequences group together as a well-defined cluster separated from the TatD DNase family, yet still closest to it among all families of the Pfam clan CL0034 (electronic supplementary material, figure S3). Therefore, in our view, EboB cannot be considered as a true member of the TatD-related DNase family, and the functional annotation of the latter cannot be applied to the former. It is likely that EboB does function as a hydrolase, which is the typical biochemical activity of members of the metallo-dependent hydrolase (amidohydrolase) superfamily. Nevertheless, it should be noted that some members of the superfamily catalyse different reactions, including decarboxylation [72] or isomerization [73].

Similarly, little can be deduced by *in silico* approaches concerning the biochemical function of EboE. Two different specific annotations (other than ‘hypothetical proteins’) are attached to EboE records in the NCBI nr protein database, ‘xylose isomerase’ or ‘AP endonuclease’. These annotations are apparently related to the fact that for a few EboE sequences, Pfam search gave marginally significant hits to the Pfam family PF01261, labelled ‘AP_endonuc_2’ and referred to as ‘Xylose isomerase-like TIM barrel’ in a more detailed annotation (see http://pfam.xfam.org/family/PF01261.22). Searching the SUPERFAMILY database assigned EboE to the Xylose isomerase-like superfamily, although not to a particular family, as the match to the most similar family defined in the database, Endonuclease IV, exhibited too high E-values. Consistent with these results, HHsearch retrieved various TIM-barrel proteins as best and significant hits for EboE. Altogether, this suggests that EboE is a TIM-barrel protein representing the Xylose isomerase-like (super)family. However, this protein group is rather diverse in regard to a specific biochemical activity. It includes isomerases of various sugars, epimerases, endonucleases, dehydratases and even putative oxygenases (see the list of the family members with solved three-dimensional structures in the Pfam database, http://pfam.xfam.org/family/PF01261.22#tabview=tab9). Hence, at the moment, the substrate(s) and the type of enzymatic reaction catalysed by EboE cannot be inferred.

The most enigmatic among all Ebo proteins is EboA. Most EboA homologues in the NCBI database are annotated as hypothetical proteins, except those from various Actinobacteria (identified by iterative PSI-blast searches) annotated as ‘sugar phosphate isomerase’ or ‘xylose isomerase’. However, these entries turned out to be hybrid proteins consisting of a C-terminal region corresponding to EboA fused to an N-terminal region representing a member of the xylose isomerase-like superfamily (data not shown). No assignment to a previously defined conserved family or domain was made for EboA proteins by searching the Pfam or the SUPERFAMILY databases. No significant similarity to other proteins was detected even when the highly sensitive HHpred search based on HMM–HMM comparisons was used. EboA proteins have no predicted transmembrane domains or an N-terminal signal peptide, indicating that they are probably cytosolic (or stromal in case of eustigmatophyte plastids) proteins. Direct biochemical characterization is necessary to unveil what the EboA proteins do in the cells.

### 3.5. Independent acquisition of *eboF* homologues by several different eukaryotic lineages

While checking the evolutionary origin of the eustigmatophyte *ebo* operon, we were interested to investigate whether it might have been acquired independently by other eukaryotic organisms. Blastp searches against eukaryotic proteins in the NCBI nr database retrieved significantly similar (in the case of EboA and EboE) or only distantly related, apparently non-orthologous (in the case of EboB, EboC, EboD; data not shown), hits. The only exception was EboF, for which four closely related eukaryotic hits were found, coming from the red alga *Chondrus crispus*, the plant parasite *Plasmodiophora brassicae* (Rhizaria), the brown alga *Ectocarpus siliculosus* and a partial protein sequence from the amoebozoan *Vermamoeba vermiformis* (electronic supplementary material, table S3). The authenticity of the *V. vermiformis* sequence could not be verified, as it is derived from a transcriptome sequencing project and bacterial contamination cannot be ruled out. However, the remaining three protein sequences corresponded to genes in nuclear genome sequences of the respective species. Inspection of the corresponding genomic loci confirmed the presence of eukaryotic introns or that the flanking genes were of a clearly eukaryotic provenance (data not shown). This observation prompted us to look for eukaryotic *eboF* homologues more systematically. The electronic supplementary material, table S3 lists putative eukaryotic *eboF* genes that we identified in genome or transcriptome sequences of various eukaryotes, specifically different protists. The corresponding predicted protein sequences do not exhibit apparent N-terminal targeting sequences (such as signal or transit peptides), suggesting they most probably function in the cytosol.

**Figure 8.**
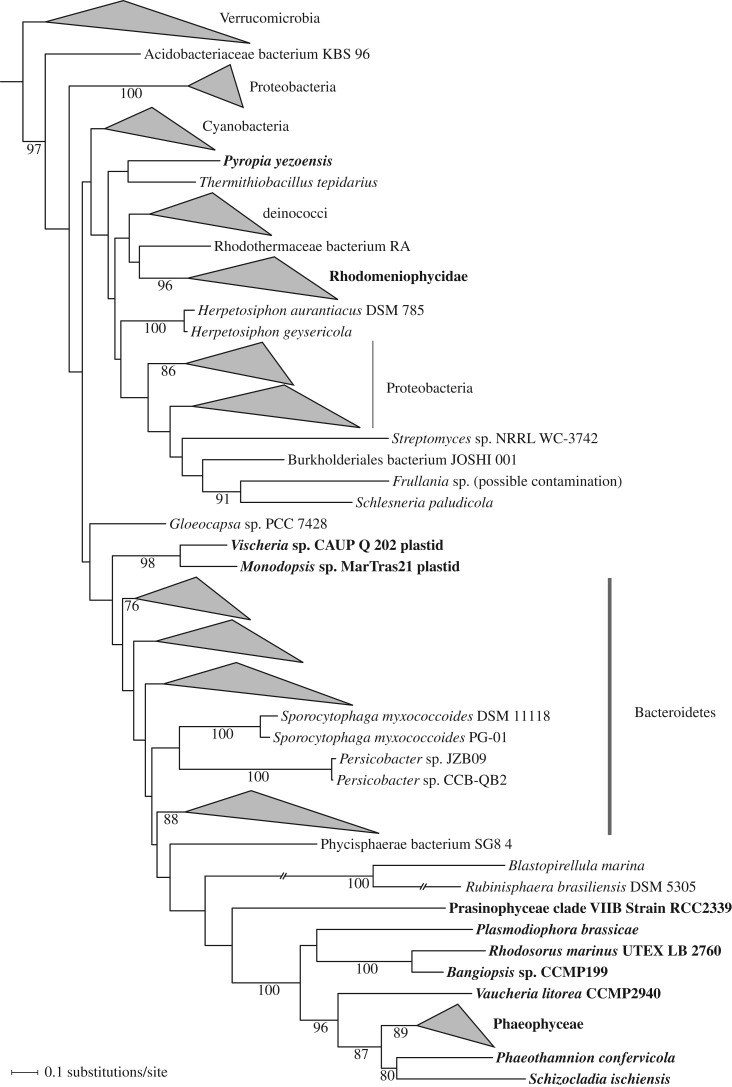
Multiple gains of the *eboF* gene by eukaryotes via HGT from bacteria. The phylogenetic tree displayed was inferred with the ML method and the LG4X+Γ substitution model from an alignment of EboF protein sequences from eukaryotes and their most similar homologues from bacteria (380 aligned amino acids). Bootstrap support values are shown at branches when more than 75%. Eukaryotic taxa are printed in bold (except the sequence from the liverwort *Frullania* sp., which is a likely bacterial contamination, see §3.5). The two branches crossed by a double slash were shortened to half of their actual length to make the figure more compact. Accession numbers of all the sequences used to infer the tree are provided in the electronic supplementary material, table S3.

To illuminate the evolutionary history of the eukaryotic *eboF* genes, we carried out a broad phylogenetic analysis of EboF protein sequences (figure 8). It suggested that the *eboF* gene was acquired by at least four different eukaryotic lineages from different bacterial donors. In one case, the *eboF* gene was gained as a part of the whole *ebo* operon by the plastome of eustigmatophytes. The other instance is an apparent HGT event from a bacterium to a nuclear genome of a red alga that seems to have occurred in the Florideophyceae class before radiation of the subclass Rhodymeniophycidae. Probably, an independent acquisition occurred in the sister lineage of Florideophyceae, i.e. the class Bangiophyceae, documented by the presence of an *eboF* homologue in the nuclear genome of *Pyropia yezoensis*. Interestingly, *eboF* homologues were encountered in transcriptome sequences of the members of a third red algal class, Stylonematophyceae. These form part of a separate, broader EboF clade that also includes sequences from two unrelated eukaryotic groups, the so-called PX clade of ochrophyte algae (Phaeophyceae, *Schizocladia ischiensis*, *Phaeothamnion confervicola*, *Vaucheria litorea*) and the rhizarian *P. brassicae* (figure 8). An EboF sequence from strain RCC2339 (an unidentified organism that appears to be a green alga representing the ‘prasinophyte’ clade VIIB) may also be affiliated with this broader eukaryotic clade, but statistical support for this is lacking and the authenticity of this sequence is uncertain, as it is derived from a large transcriptome project known to be plagued with contaminations [74]. The final eukaryotic EboF sequence in our analysis came from a transcriptome assembly of the liverwort *Frullania* sp. (figure 8). This is a sole *eboF* homologue identified in all plant sequence resources investigated, so we consider it a likely contamination from a bacterium.

Although the interpretation of the occurrence of *eboF* homologues in different eukaryotes as results of HGT events from bacteria is undisputed, our analysis did not reveal the exact source lineages for these transfers. As discussed above for the *ebo*F genes in eustigmatophyte plastomes, this is a common situation in the field of HGT research. The phylogenetically disparate composition of the ‘main’ eukaryotic EboF clade may look intriguing, but a similar pattern has frequently been observed for eukaryotic metabolic genes acquired from bacteria. For example, genes for squalene-tetrahymanol cyclase exhibit an extremely patchy distribution in phylogenetically diverse eukaryotes, yet all these genes constitute a strongly supported exclusively eukaryotic clade [75]. One possible explanation for such cases is an ancient acquisition of the gene by an early eukaryote and extensive multiple losses from most descendants of this ancestor. Alternatively, more recent acquisition of the gene by a particular eukaryotic lineage followed by further dissemination among eukaryotes via HGT between different eukaryotic lineages is also conceivable. In some cases, selective forces for such spreading may be readily envisaged. For example, the distribution of the aforementioned squalene-tetrahymanol cyclase is obviously related to multiple independent emergences of an anaerobic lifestyle in different eukaryotes. At the moment, discussing such scenarios is not possible for EboF, as its exact biochemical function remains unknown (see §3.4.3). However, as the non-eustigmatophyte eukaryotes with *eboF* apparently lack the other five *ebo* genes, our analysis indicates that EboF does not functionally depend on the other Ebo proteins.

### 3.6. Is the *ebo* operon involved in the synthesis of scytonemin?

While surveying the literature for previous reports on *ebo* genes, we encountered a work that touches upon the possible biochemical role of the *ebo* operon. Soule and colleagues studied the genomic context of a cluster of six genes denoted *scyA* to *scyF* and conserved in some cyanobacteria, which had been implicated in the production of the natural sunscreen scytonemin [76]. They noted that the genes downstream of this cluster are also generally conserved in the identity and relative position between the cyanobacteria studied. Importantly for our case, six of these genes correspond to *eboA* through *eboF*. Soule *et al*. assigned functional annotation to some of them, specifically ‘hydrolase’ for *eboB*, ‘UbiA’ for *eboC*, ‘AroB’ for *eboD* and ‘phosphodiesterase’ for *eboF*. This is partly congruent with our analyses above, but note that in the case of *eboC* and *eboD* the annotation provided by Soule *et al*. is misleading, as we have shown EboB is not a UbiA orthologue, but a novel enzyme with unknown substrate specificity in the UbiA superfamily, and EboD is not an AroB (i.e. 3-dehydroquinate synthase) orthologue, but a novel lineage within the same superfamily probably with a different catalytic activity from AroB (DHQS). Hence, we do not support the conjecture of Soule *et al*. that their scytonemin cluster-associated ‘AroB’ is involved in the biosynthesis of aromatic amino acids.

The enzymes responsible for catalysing the initial reactions of scytonemin biosynthesis have been identified and are encoded by *scyABC* genes [77,78], but the enzymes involved in the last two reactions, specifically the penultimate oxidation and the final dimerization step of a monomeric scytonemin moiety, remain uncharacterized. Recently, Ferreira & Garcia-Pichel [79] tested the possibility that products of the *scyDEF* in the cyanobacterium *Nostoc punctiforme* may be the missing enzymes, but their results ruled out this hypothesis. Thus, the authors speculated that the final reactions of scytonemin biosynthesis might possibly be catalysed by proteins encoded by the ‘satellite five-gene cluster’ (Npun_F5232 to Npun_F5236). This cluster, in fact, corresponds to the *eboA*, *eboB*, *eboC*, *eboE* and *eboF* genes in *N. punctiforme*. In contrast to the majority of other cyanobacteria with the scytonemin cluster, *N. punctiforme* has retained only an *eboD* gene (‘AroB’ *sensu* Soule *et al*.) directly downstream of the *scy* region, whereas the remaining five *ebo* genes are present elsewhere in the genome together with a second *eboD* paralogue located in the reverse orientation upstream of the *eboA* gene (figure 3). Interestingly, it was previously shown that expression of the satellite cluster in *N. punctiforme* is induced by UV exposure [80], which supports the hypothesis about its functional link to scytonemin production.

However, scytonemin is restricted to Cyanobacteria [79,81], whereas our analyses have established that the *ebo* operon is present in many different bacterial groups. Hence, if there is any role of the operon in scytonemin biosynthesis, it would mean that the involved Ebo proteins either have a broader specificity allowing interaction with different substrates, or that the specificity has differentiated between Cyanobacteria and other groups such that the *ebo* operon is involved in the production of different compounds in different organisms. In addition, the molecular structure of scytonemin and the nature of the reactions Ferreira & Garcia-Pichel [79] hypothesized to be possibly catalysed by enzymes of the *ebo* operon do not obviously fit the enzymatic activities predicted for at least some of the Ebo proteins, such as EboC, EboD and EboF. Therefore, we believe that direct involvement of these proteins in scytonemin synthesis is unlikely and they must be involved in catalysing the synthesis of a separate compound. However, we cannot exclude the possibility of their indirect role, e.g. in synthesizing a cofactor employed by *bona fide* scytonemin–synthesis enzymes.

### 3.7. The *ebo* operon most probably mediates the synthesis of an isoprenoid–cyclitol-derived compound

The presence of a new member of the SPC superfamily—EboD, possibly using sedoheptulose 7-phosphate as a substrate—suggests that the *ebo* operon may be involved in the synthesis of a cyclitol-derived secondary metabolite. Cyclitol-derived compounds are an extremely diverse group of chemicals and the metabolic pathways mediating their production differ accordingly. Interestingly, at least one previously characterized example includes a reaction catalysed by a member of the UbiA superfamily, which also includes the EboC protein (see §3.4.1). This specifically concerns the product called BE-40644, isolated from the actinobacterium *Actinoplanes* sp. A40644 and shown to have an inhibitory effect on the human thioredoxin system [82]. It is synthesized from sedoheptulose 7-phosphate by first converting it to 2-epi-5-epi-valiolone by the enzyme EEVS encoded by one of the genes of the BE-40644 synthesis gene cluster [66,83]. After a series of further modifications, a particular intermediate is prenylated (farnesylated) presumably by a prenyltransferase encoded by another gene of the same cluster [84], which, in fact, is a member of the UbiA superfamily (other than EboC; data not shown). Therefore, we posit that EboD and EboC similarly cooperate to make a hybrid isoprenoid–cyclitol substance.

The presence of EboF homologues in various eukaryotes independently of the other five *ebo* genes (see §3.5) suggests that the putative phosphate moiety-containing substrate of this predicted hydrolase (see §3.4.3) may be a commonly occurring metabolite, not a specific product of the action of Ebo proteins. Indeed, although SPC superfamily enzymes use a phosphorylated sugar as a substrate, the phosphate moiety is extracted from the carbohydrate skeleton during the reaction, and the resulting cyclitol molecule lacks a phosphate group [51,66]. Similarly, a prenyl diphosphate is a typical substrate of UbiA superfamily enzymes, but the diphosphate moiety is liberated in the reaction and does not become a part of the prenylated product [50]. EboF thus functions either in a separate branch of the hypothetical pathway that makes an intermediate subsequently condensed with a product of the branch including the EboD (and possibly also EboC) step, or it works upstream of EboD or EboC. Which enzyme would mediate the condensation step assumed by the first possibility? EboB and EboE are predicted to exhibit hydrolytic, isomerization, dehydration or decarboxylation activities (see §3.4.4), so they do not seem likely to catalyse a step that would join together products of converging branches of our hypothetical biosynthetic pathway. We cannot exclude the possibility that the enigmatic EboA is responsible for catalysing such a condensation step. However, given the existence of an alternative version of the *ebo* operon with the apparently unrelated EboG in place of EboA, we assume that these small rapidly evolving proteins are more likely to serve as regulatory subunits of another Ebo protein or are involved in the terminal step of the pathway leading to alternative products depending on the presence of EboA or EboG.

These considerations suggest that the pathway in question may have only one step in which two separate organic molecules are fused together—the one catalysed by the putative prenyltransferase EboC. Then EboF would work upstream of EboD or EboC. Above we suggested that sedoheptulose 7-phosphate, a direct product of the pentose or the Calvin cycle, is a likely substrate of EboD, but we cannot dismiss the possibility that another phosphorylated sugar is used instead. This alternative substrate then could be produced by EboF, e.g. by dephosphorylating a bisphosphate precursor or liberating the sugar phosphate by hydrolysing a phosphodiester or phosphoanhydride bond in a larger molecule. A role of EboF in preparing a substrate for EboC is also conceivable. Although most UbiA superfamily members use prenyl diphosphates as their substrate, at least one member, decaprenylphosphoryl-5-phosphoribose (DPPR) synthase, uses a prenyl phosphate (specifically decaprenyl phosphate [50]), which may be true also for EboC. Because prenyl chains of different length are originally synthesized as diphosphates [85], a specific phosphatase is needed to produce a prenyl phosphate (such as has been described, e.g. for the synthesis of undecaprenyl phosphate [86]). EboF could thus hypothetically be a prenyl diphosphate phosphatase.

The position of steps catalysed by EboB and EboE in the pathway encoded by the *ebo* operon remains similarly hypothetical. These enzymes may be involved in the synthesis of the putative EboC substrate by specifically modifying a prenyl chain produced by a common prenyl synthesis pathway, or they may mediate formation of the EboD substrate (in the case the substrate is in fact not the apparently most likely candidate, sedoheptulose 7-phosphate). Alternatively, they may be responsible for modifications of the cyclitol produced by EboD, or they may operate downstream of the EboC-catalysed step by further modifying the putative isoprenoid–cyclitol compound. Indeed, the synthesis of BE-40644, the isoprenoid–cyclitol-derived metabolite mentioned above, includes dehydration of the cyclitol unit and final cyclization of the farnesyl chain via intramolecular rearrangement (isomerization) [66,84]. Such enzymatic steps are reminiscent of reactions catalysed by superfamilies accommodating EboB and EboE (see §3.4.4). Although our reasoning on the role of the *ebo* operon and individual Ebo proteins includes some uncertainty, we believe it provides a good basis for future direct biochemical investigations.

## 4. Conclusion

In this study, we have described a peculiar case of a HGT event of a whole operon from a bacterium of the phylum Bacteroidetes into the plastid genome of an early eustigmatophyte alga before the split of the *Vischeria* and *Monodopsis* lineages. A molecular clock analysis based on the 18S rRNA gene phylogeny of ochrophyte algae estimated the divergence of the genera *Vischeria* and *Monodopsis* to have most probably occurred around 120 Ma [87]. Hence, the *ebo* operon is apparently functional and confers a selective advantage to its bearers, as it has survived intact in eustigmatophyte plastomes for such a long time. Nevertheless, the *ebo* operon is secondarily missing from plastomes of the genus *Nannochloropsis* (including *Microchloropsis*), indicating that it did not become essential for eustigmatophytes and specific conditions or evolutionary circumstances enabled its loss (figure 1). Defining the causes behind the phylogenetic distribution of the *ebo* operon in eustigmatophytes and bacteria must wait until the biochemical pathway executed by the six conserved Ebo proteins is studied by direct *in vitro* or *in vivo* approaches, and its final product is characterized. Our sequence analyses of individual Ebo proteins provide a good starting point for these future investigations. In analogy with secondary metabolite gene clusters described before, we speculate that this unknown product might have interesting bioactive properties, serving, for example, as an antimicrobial substance or a protectant against adverse environmental factors.

While researchers have mastered artificial methods for engineering plastid genomes to introduce novel metabolic capacities into the plastid [88], such a case of natural engineering we have discovered in eustigmatophytes, to our knowledge, has not been reported before. It makes these eustigmatophytes an even more exciting target for dedicated research than ever before.

## Supplementary Material

Supplementary figures “The plastid genome of some eustigmatophyte algae harbours a bacteria-derived six-gene cluster for biosynthesis of a novel secondary metabolite” by Tatiana Yurchenko et al

## Supplementary Material

Supplementary tables “The plastid genome of some eustigmatophyte algae harbours a bacteria-derived six-gene cluster for biosynthesis of a novel secondary metabolite” by Tatiana Yurchenko et al

## Data accessibility

The newly sequenced plastomes were deposited at GenBank with accession numbers KX839261.1 (*Vischeria* sp. CAUP Q 202) and KX839260.1 (*Monodopsis* sp. MarTras21). The datasets supporting this article have been uploaded as part of the electronic supplementary material.
