# Supplementary material for: The plastid genome of some eustigmatophyte algae harbours a bacteria-derived six-gene cluster for biosynthesis of a novel secondary metabolite
Source: Open Biol. 2016 Nov 30;6(11):160249. doi: 10.1098/rsob.160249 (PMC5133447; doi:10.1098/rsob.160249)
Supplement: Supplementary figures “The plastid genome of some eustigmatophyte algae harbours a bacteria-derived six-gene cluster for biosynthesis of a novel secondary metabolite” by Tatiana Yurchenko et al [file rsob160249supp1.pdf]

Supplementary data file to the paper

“Yurchenko T, Ševčíková T, Strnad H, Butenko A, Eliáš M (2016) The plastid genome of some eustigmatophyte algae harbours a bacteria-derived six-gene cluster for biosynthesis of a novel secondary metabolite. *Open Biology* **6**: 160249. http://dx.doi.org/10.1098/rsob.160249”

Supplementary figures S1 to S3

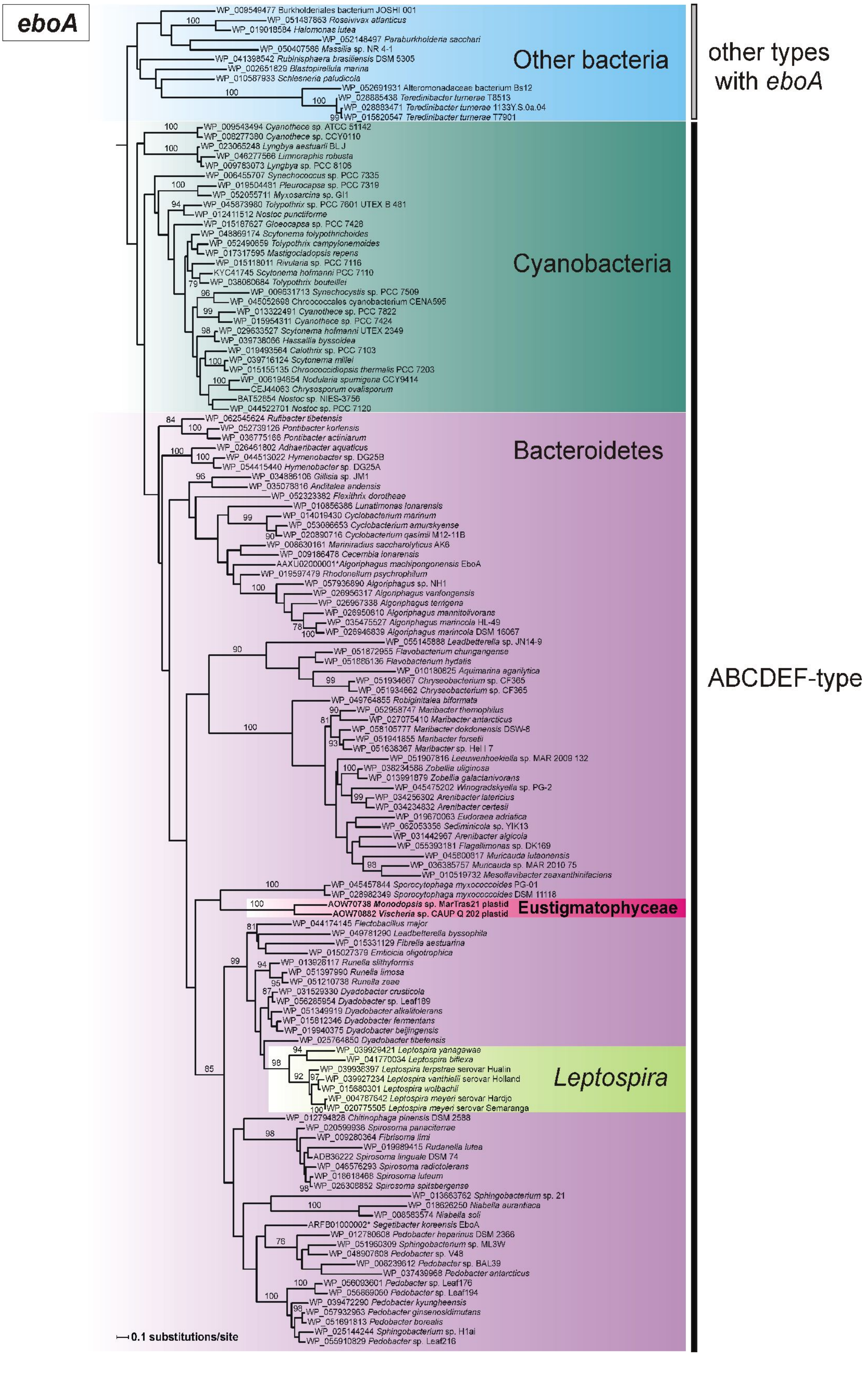

Figure S1. Phylogenetic analyses of individual Ebo proteins aimed at deducing the origin of the eustigmatophyte *ebo* operon. The trees were inferred using RAxML and the LG4X+Γ substitution model. Bootstrap support values are shown at branches when >75%. Five groups of species are distinguished by a different background: Bacteroidetes – violet; Cyanobacteria – blue-green; Eustigmatophyceae – red; *Leptospira* – light green; other bacteria – blue. Taxa are divided into three conveniently defined groups according to the type of the *ebo* operon they possess (see the vertical bars on the right side). Note that some species assigned in the figure to the ABCDEF-type of the operon secondarily deviate from this presumed ancestral form for the given major taxa (see section 3.2 in the main text). A phylogenetic tree for the EboC protein is shown in the main text as figure 4.

(A) Phylogenetic analysis of EboA sequences based on an alignment of 182 positions.

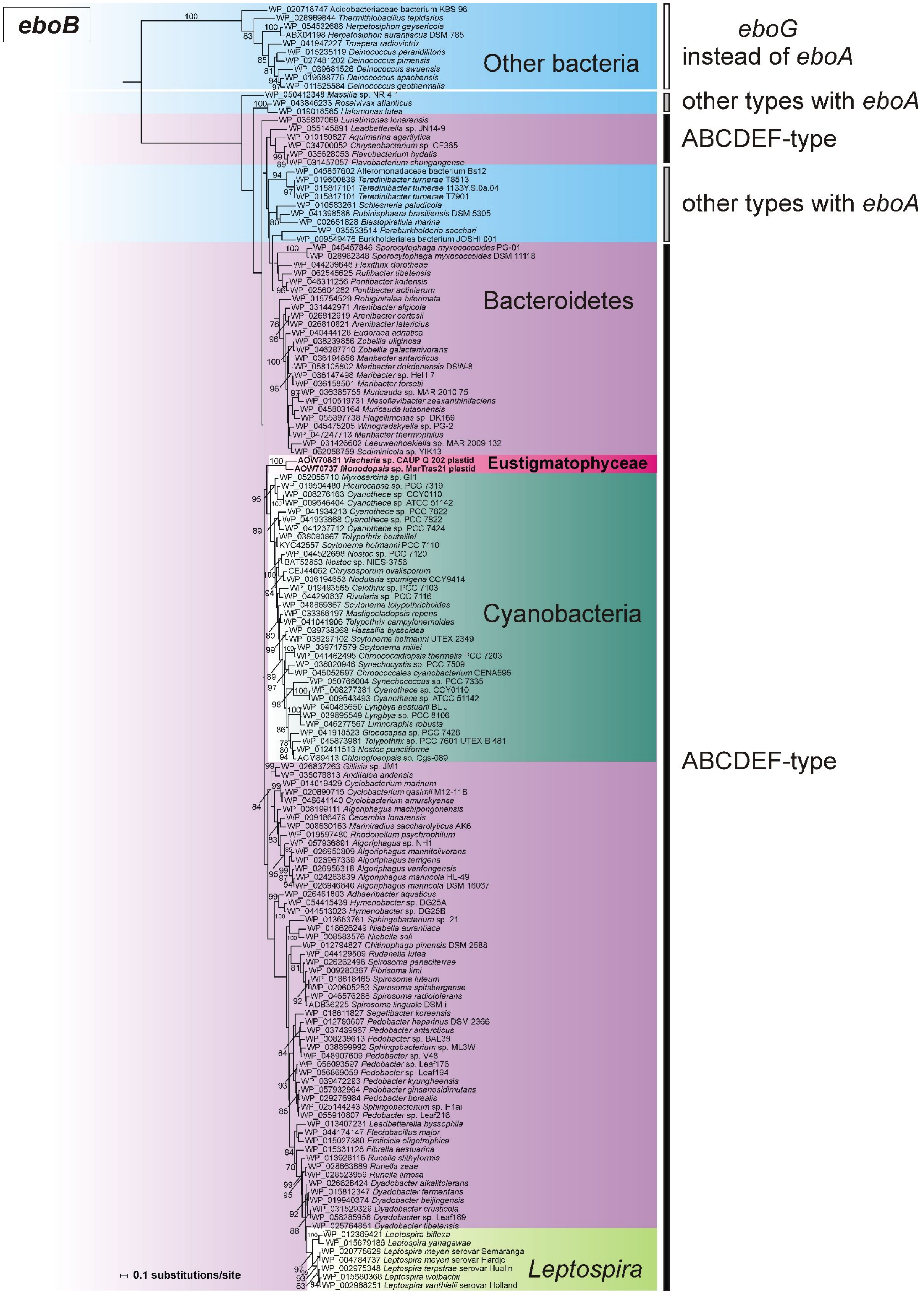

FigureS1 cont. (B) Phylogenetic analysis of EboB sequences based on an alignment of 285 positions.

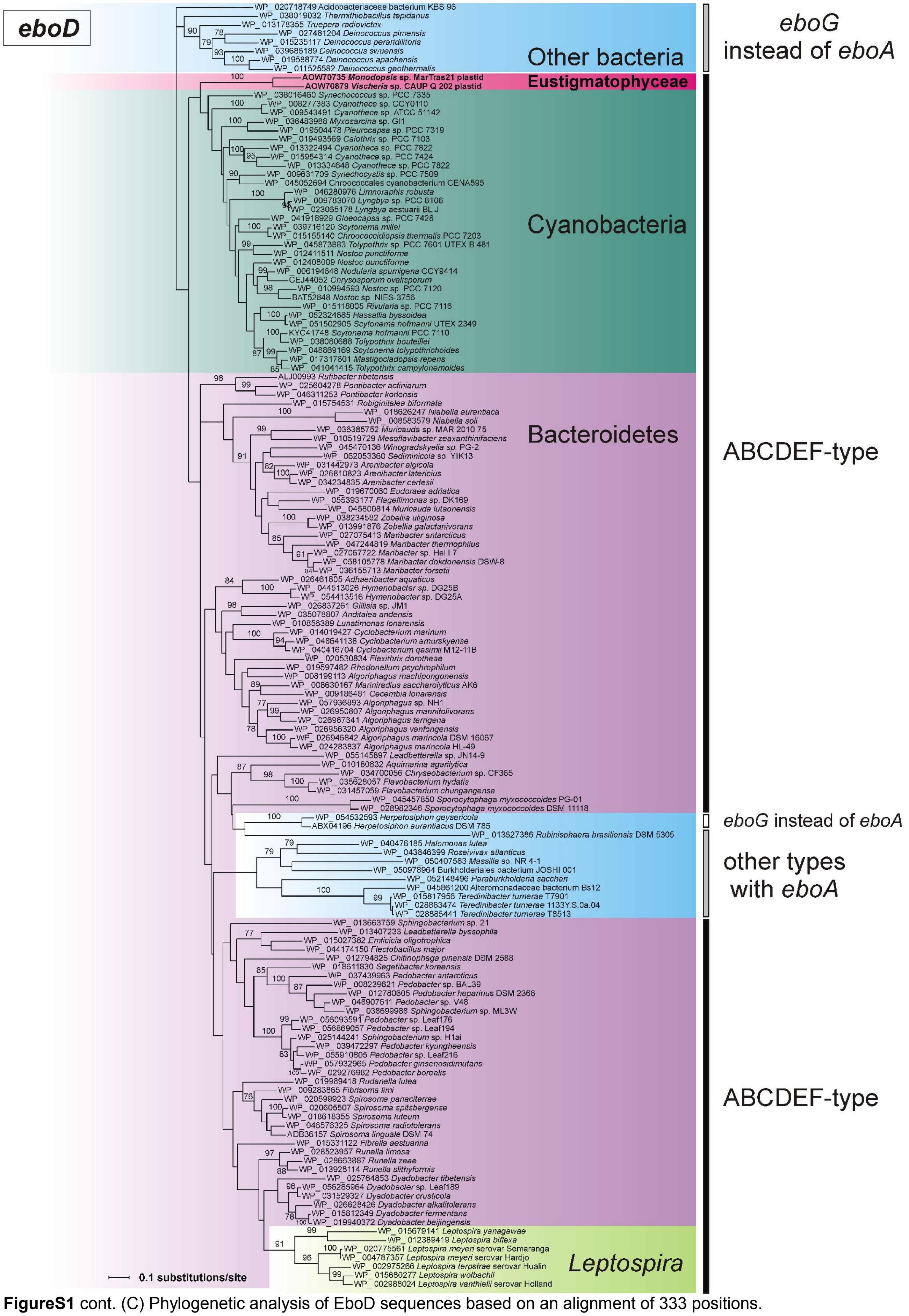

FigureS1 cont. (C) Phylogenetic analysis of EboD sequences based on an alignment of 333 positions.

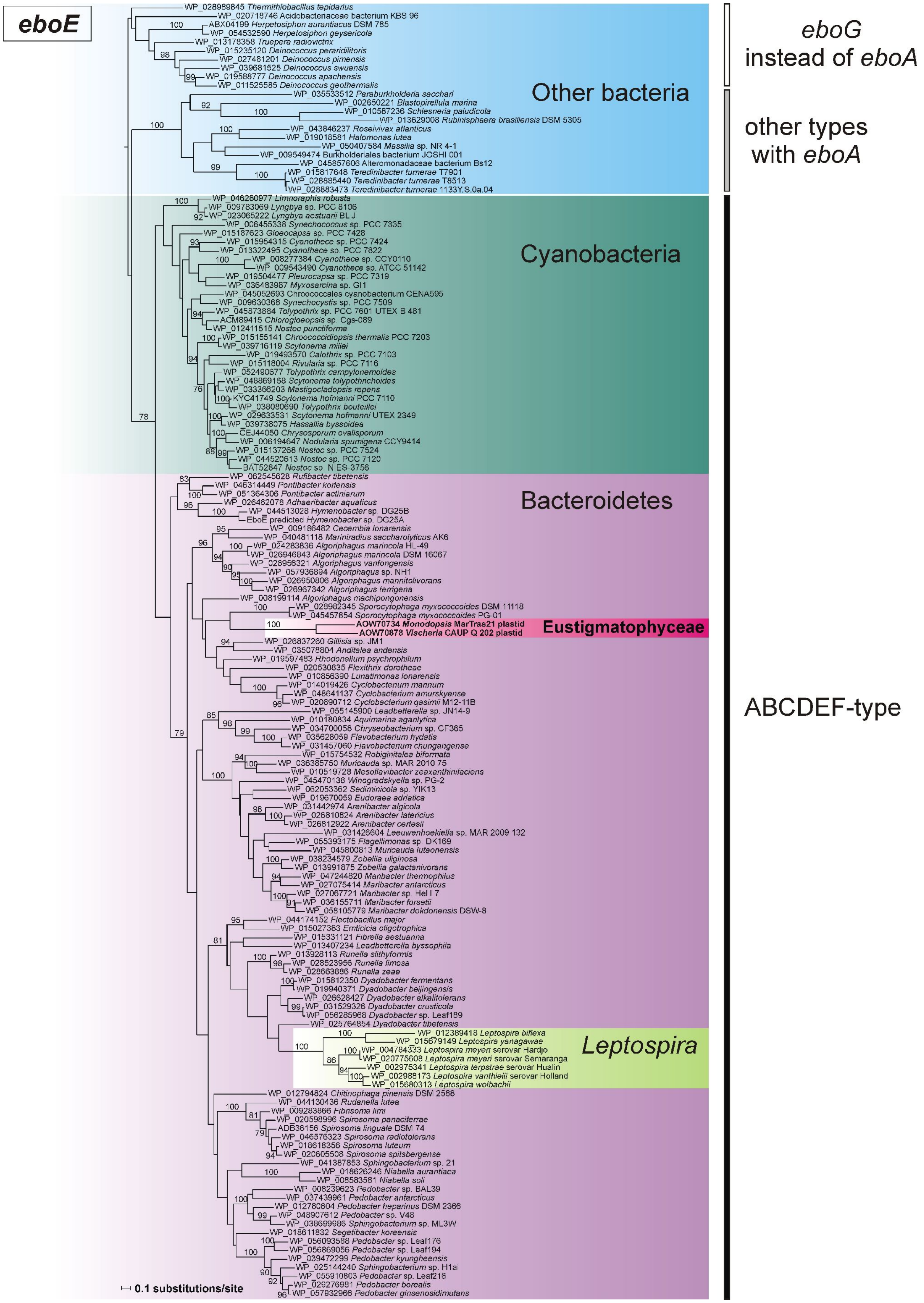

FigureS1 cont. (D) Phylogenetic analysis of EboE sequences based on an alignment of 313 positions.

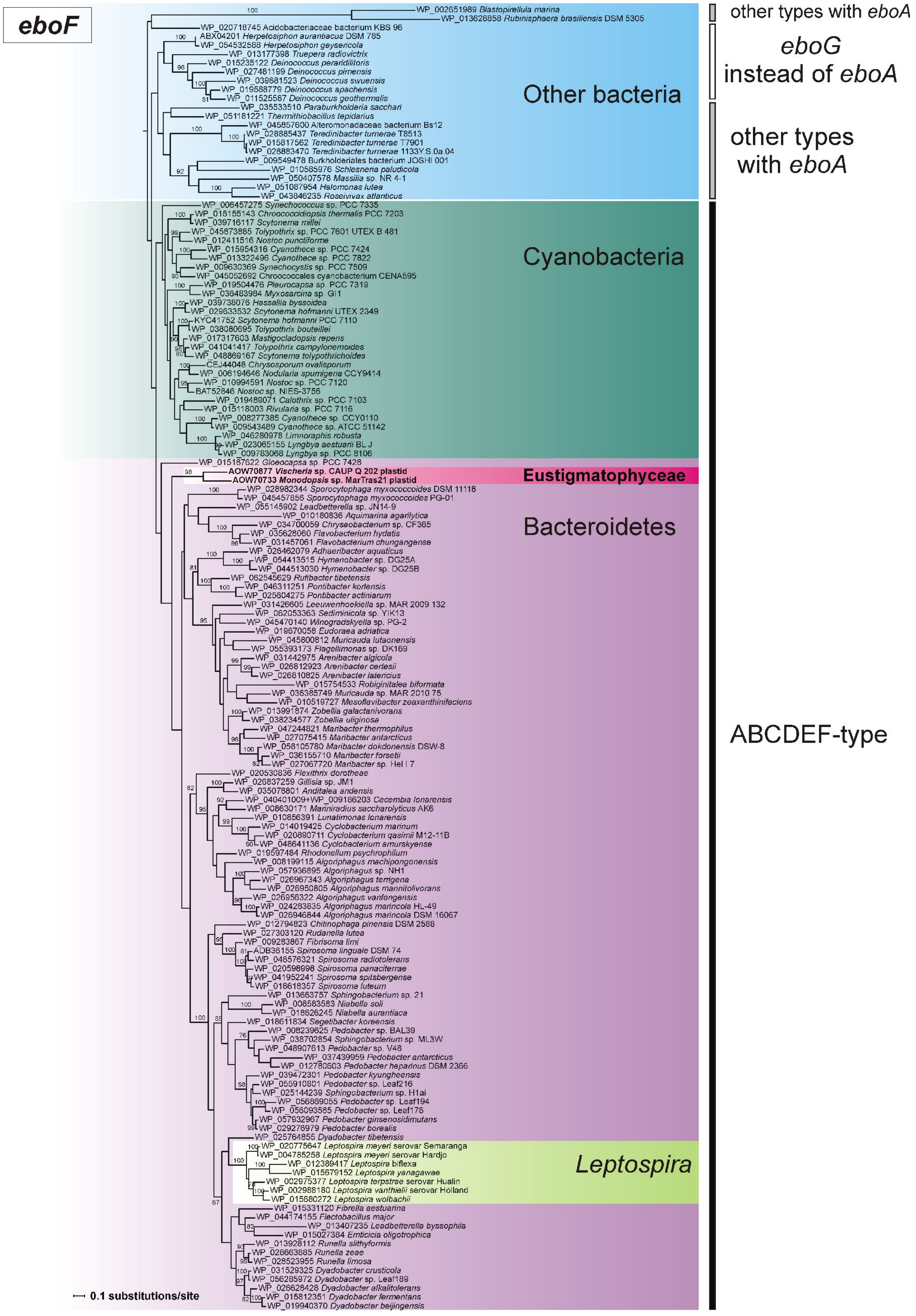

FigureS1 cont. (E) Phylogenetic analysis of EboF sequences based on an alignment of 409 positions.

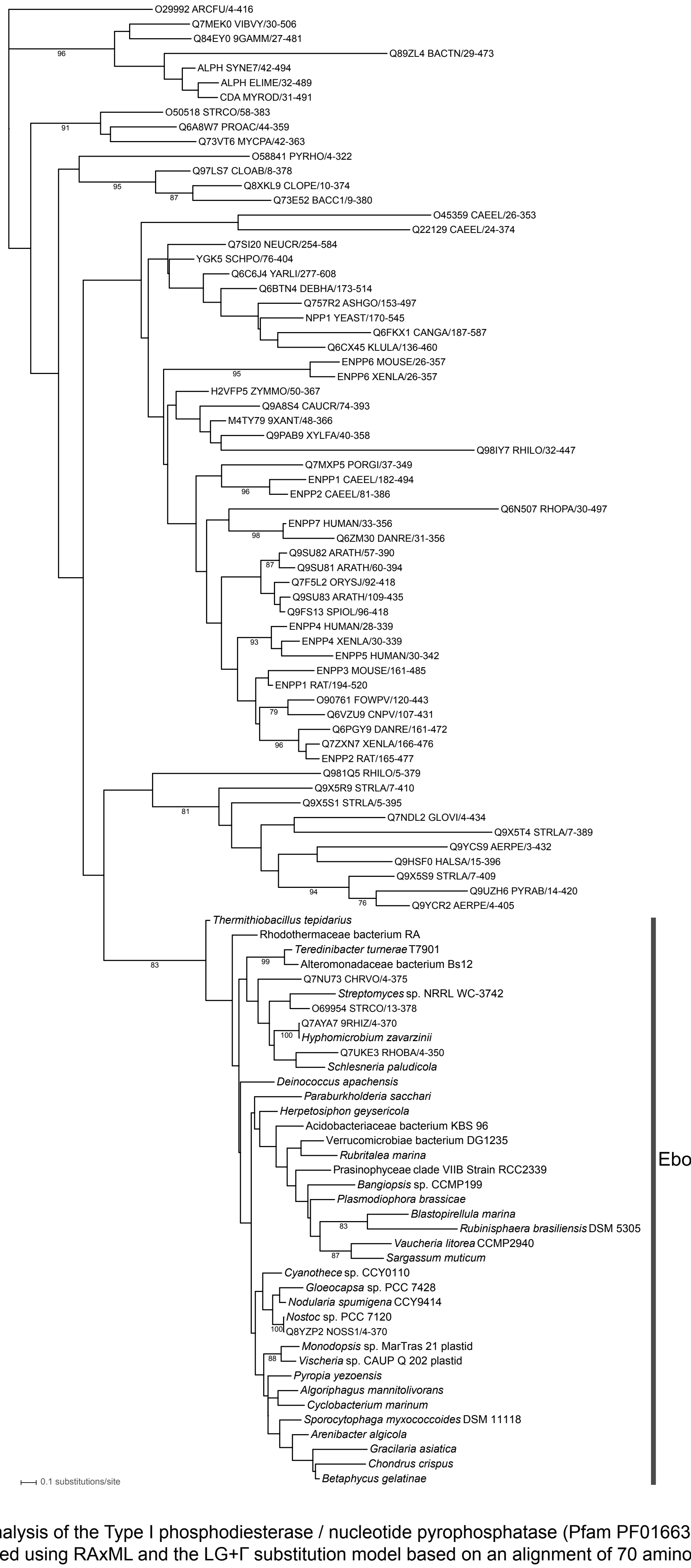

Figure S2. Phylogenetic analysis of the Type I phosphodiesterase / nucleotide pyrophosphatase (Pfam PF01663) family, including EboF. The trees were inferred using RAxML and the LG+Γ substitution model based on an alignment of 70 amino acid positions. Bootstrap support values are shown at branches when >75%. GenBank accesion numbers of EboF sequences indicated with taxon names are provided in Table S2. The remaining sequences come from the seed alignment of the PF01663 family as provided by the Pfam database and are indicated with their UniProtKB locus names.

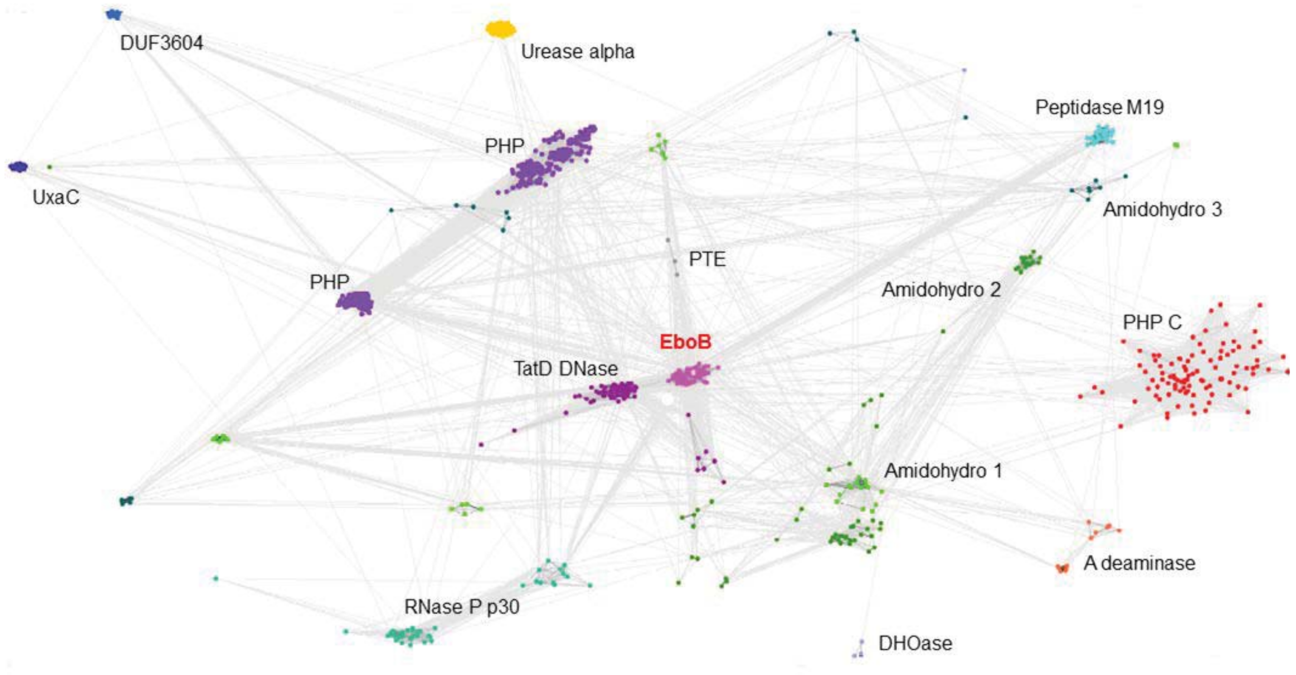

Figure S3. Cluster analysis of the Amidohydrolase superfamily (Pfam clan CL0034) showing EboB (in pink) as a separate cluster most similar ot the TatD DNase family (in violet). The analysis was performed as described in detail in Material and Methods (section 2.5).
